# Flagellin hypervariable region determines symbiotic properties of commensal *Escherichia coli* strains

**DOI:** 10.1371/journal.pbio.3000334

**Published:** 2019-06-17

**Authors:** Alex Steimle, Sarah Menz, Annika Bender, Brianna Ball, Alexander N. R. Weber, Thomas Hagemann, Anna Lange, Jan K. Maerz, Raphael Parusel, Lena Michaelis, Andrea Schäfer, Hans Yao, Hanna-Christine Löw, Sina Beier, Mehari Tesfazgi Mebrhatu, Kerstin Gronbach, Samuel Wagner, David Voehringer, Martin Schaller, Birgit Fehrenbacher, Ingo B. Autenrieth, Tobias A. Oelschlaeger, Julia-Stefanie Frick

**Affiliations:** 1 Institute of Medical Microbiology and Hygiene, University of Tübingen, Tübingen, Germany; 2 German Center for Infection Research, Partner Site Tübingen, Tübingen, Germany; 3 Department of Immunology, University of Tübingen, Tübingen, Germany; 4 Chair of Algorithms in Bioinformatics, Faculty of Computer Science, University of Tübingen, Tübingen, Germany; 5 Department of Infection Biology, University Hospital Erlangen, Erlangen, Germany; 6 Department of Dermatology, University Hospital Tübingen, Tübingen, Germany; 7 Institute for Molecular Infection Biology, University of Würzburg, Germany; Instituto Gulbenkian de Ciencia, PORTUGAL

## Abstract

*Escherichia coli* represents a classical intestinal gram-negative commensal. Despite this commensalism, different *E*. *coli* strains can mediate disparate immunogenic properties in a given host. Symbiotic *E*. *coli* strains such as *E*. *coli* Nissle 1917 (EcN) are attributed beneficial properties, e.g., promotion of intestinal homeostasis. Therefore, we aimed to identify molecular features derived from symbiotic bacteria that might help to develop innovative therapeutic alternatives for the treatment of intestinal immune disorders. This study was performed using the dextran sodium sulphate (DSS)-induced colitis mouse model, which is routinely used to evaluate potential therapeutics for the treatment of Inflammatory Bowel Diseases (IBDs). We focused on the analysis of flagellin structures of different *E*. *coli* strains. EcN flagellin was found to harbor a substantially longer hypervariable region (HVR) compared to other commensal *E*. *coli* strains, and this longer HVR mediated symbiotic properties through stronger activation of Toll-like receptor (TLR)5, thereby resulting in interleukin (IL)-22–mediated protection of mice against DSS-induced colitis. Furthermore, using bone-marrow–chimeric mice (BMCM), CD11c+ cells of the colonic lamina propria (LP) were identified as the main mediators of these flagellin-induced symbiotic effects. We propose flagellin from symbiotic *E*. *coli* strains as a potential therapeutic to restore intestinal immune homeostasis, e.g., for the treatment of IBD patients.

## Abstract 2

A flagellum renders bacteria motile, but this study reveals another property important for symbiosis: the hypervariable region of Escherichia coli flagellin strongly determines activation of TLR5, mediating benefits for the host such as protection against colitis.

## Introduction

*E*. *coli* belongs to the phylum of gram-negative Proteobacteria. Besides certain pathogenic strains, *E*. *coli* represents a commensal member of the intestinal microbiota. However, distinct commensal *E*. *coli* strains can mediate substantially different immunological host responses. On the one hand, so-called “pathobionts” may induce severe pathological inflammatory reactions in a certain genetically predisposed or environmentally challenged host. On the other hand, “symbionts” generally provide beneficial effects and do not induce inflammatory responses at intestinal mucosal interfaces [1]. Although *Escherichia* is usually not among the most abundant bacterial genera within a healthy, balanced, and diversified intestinal microbiota [2,3], the impact of enhanced proportions of Proteobacteria in general and *E*. *coli* strains in particular on inflammatory processes in Inflammatory Bowel Disease (IBD) patients has widely been reported [4–7]. This observation leads to questions concerning mechanistic and structural differences between symbiotic and nonsymbiotic commensal *E*. *coli* strains and their subsequent impact on IBD pathology. One of the most intensely studied symbiotic *E*. *coli* strains is *E*. *coli* Nissle 1917 (EcN). EcN is generally classified as a probiotic and is the only bacterial symbiont that is successfully used to extend remission phases in IBD patients in clinical routine [8]. In this context, EcN mediates similar therapeutic effects as mesalamine, the gold standard therapeutic to extend remission time in ulcerative colitis (UC) patients [9–11]. EcN provides different beneficial properties such as (1) the formation of biofilms [12] leading to the production of defensins [12,13], (2) strengthening of tight junctions within the intestinal epithelium [14], (3) direct antimicrobial effects via secretion of bacteriocins and microcins [15], and (4) direct interaction with the host immune system [16].

However, most of these effects are thought to require viable EcN bacteria, and the recommended therapeutic EcN dose comprises extremely high numbers of vital cells [2]. In general, administration of viable bacteria involves the risk of proactive bacterial translocation across the epithelial barrier, particularly in inflamed intestinal tissue, which provides a disturbed barrier integrity. Thus, use of live bacteria for the treatment of ongoing inflammatory reactions bears considerable risks. Therefore, it appears desirable to identify bacterial factors that distinguish symbiotic from nonsymbiotic *E*. *coli* strains. Such identified symbiotic factors could then be used as novel therapeutic approaches to restore gut immune homeostasis and could offer a wide range of potential clinical applications.

The investigation of commensal bacteria-mediated symbiotic properties requires the use of adequate mouse models. One of these mouse models involves the application of dextran sodium sulphate (DSS). Administration of DSS leads to an IBD-resembling phenotype in mice of almost all genetic backgrounds [17]. The similarities between IBD in humans and DSS-induced colitis in mice include a similar inflammatory gene expression pattern [18], T-cell accumulation in the colon [19], and the development of a chronic pathology after initial acute inflammation involving a T helper (Th)1/Th2 cytokine secretion pattern [20]. As in IBD patients, DSS-induced colitis in mice leads to influx and transepithelial migration of neutrophils into the mucosal epithelium and lumen, leading to the formation of crypt abscesses [21–24]. Furthermore, DSS-induced colitis in mice provides comparable sensitivity toward therapeutics as in IBD [25], making this model appropriate for preclinical studies involving the evaluation of new potential treatments for IBD patients [26], e.g., commensal-derived molecular features such as surface-associated structures.

Various bacterial surface structures serve as so-called microbe-associated molecular patterns (MAMPs), molecules that are recognized by host pattern-recognition receptors (PRRs), e.g., Toll-like receptors (TLRs) [27]. PRR sensing leads to activation of the innate and modulation of the adaptive immune system. One of these MAMPs is flagellin, the constitutive protein building up bacterial flagella. Flagellin typically consists of different structural domains: all flagellins contain N-terminal and C-terminal D0 and D1 domains that form the structurally highly homogenous so-called “conserved region” [28–30]. Some flagellins, such as most *E*. *coli* flagellins, additionally contain a “hypervariable region” (HVR) made up of C- and N-terminal D2 domains as well as of a central D3 domain [28]. Extracellular flagellin is recognized only as a monomer [29] by host TLR5 [30], leading to myeloid differentiation primary response (MyD)88-dependent activation of nuclear factor “kappa-light-chain–enhancer” of activated B-cells (NFκB) [31,32]. Interestingly, flagellin represents a major target antigen in human IBD patients as well as in experimental mouse models for colitis [33]. Additionally, certain *TLR5* SNPs are associated with higher incidence of UC [34] and colon cancer [35]. Furthermore, TLR5-deficient mice are prone to develop spontaneous intestinal inflammation [36], and intestinal TLR5 signaling was demonstrated to be crucial for preventing gut inflammation and metabolic syndrome in mice [37].

Taking these data together, flagellin recognition by intestinal TLR5-expressing host cells seems to be crucially involved in shaping host immunity, leading to maintenance of intestinal immune homeostasis. Therefore, we hypothesized that the precise flagellin structure could be a decisive factor that allows for a distinction between symbiotic and nonsymbiotic *E*. *coli* strains. Here, we demonstrate that EcN flagellin is sufficient to mediate a crucial part of EcN’s symbiotic properties via TLR5 on intestinal CD11c^+^ cells within the lamina propria (LP). Furthermore, we provide evidence that the structure of the flagellin HVR is a decisive factor that classifies EcN as a symbiont. In consequence, symbiotic HVR containing flagellin structures might be used as innovative therapeutic approaches for the treatment of IBD.

## Results

### Interaction of EcN flagella with host TLR5 is crucial for the beneficial effects of EcN during DSS-induced colitis

**Fig 1:**
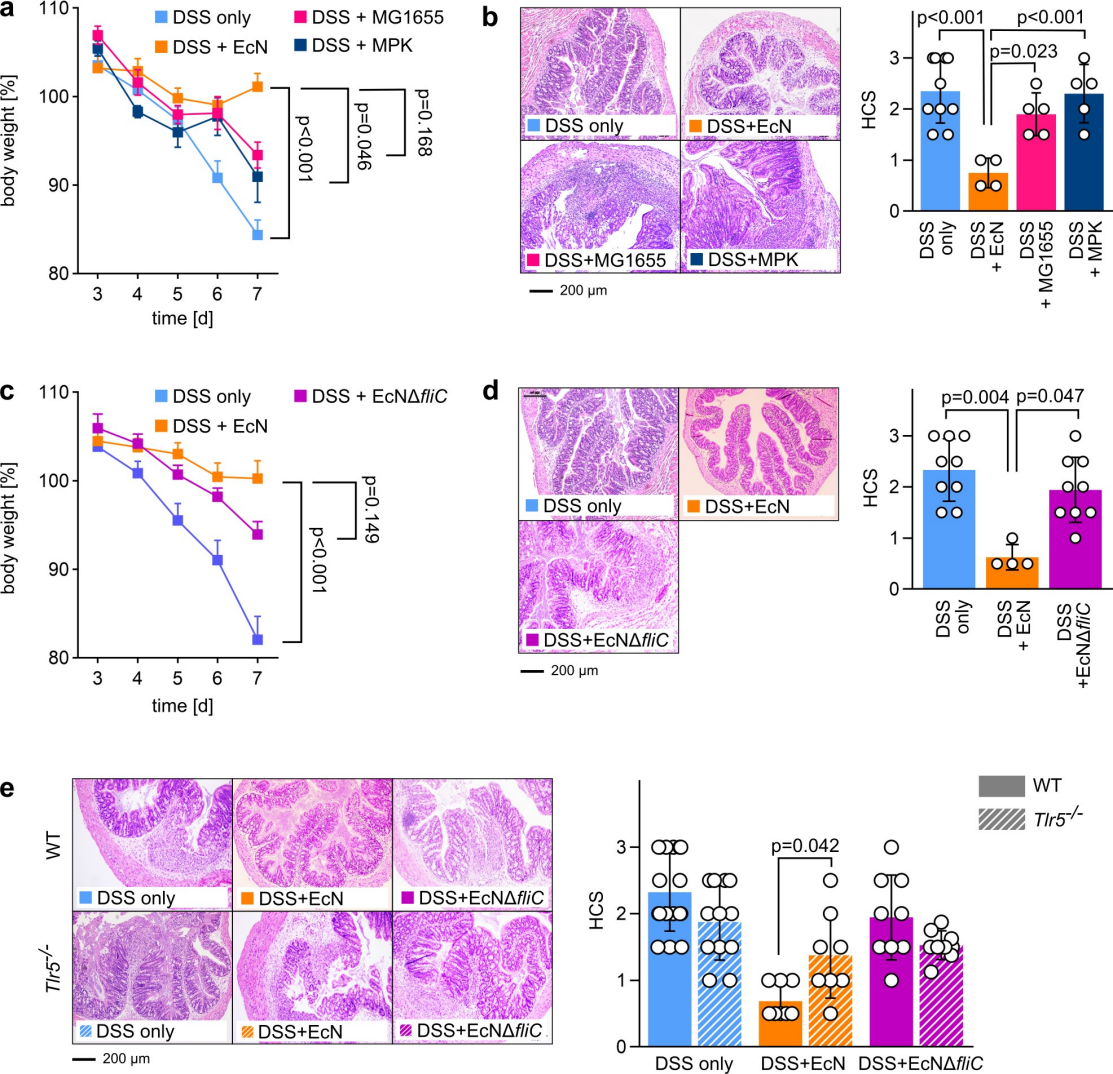
Influence of different commensal *E*. *coli* strains on DSS-induced colitis in WT and *Tlr5*^−/−^ mice. (a + b) SPF C57BL/6 WT mice aged 6 to 8 weeks were administered 3.5% DSS in drinking water at day 0. Mice were additionally treated with EcN (DSS + EcN), MG1655 (DSS + MG1655), or MPK (DSS + MPK) resuspended in DSS-containing drinking water at 10^8^ bacteria mL^−1^. (a) Change in body weight relative to start of DSS administration at day 0. (b) Left panel: HE-stained colonic sections at day 7 after start of DSS administration. Right panel: HCS at day 7. (c + d) SPF C57BL/6 WT mice aged 6 to 8 weeks were administered 3.5% DSS in drinking water at day 0. Mice were additionally treated with EcN (DSS + EcN) or an EcNΔ*fliC* deletion mutant (DSS + EcNΔ*fliC*) resuspended in DSS-containing drinking water at 10^8^ bacteria mL^−1^. (c) Change in body weight relative to start of DSS administration at day 0. (d) Left panel: HE-stained colonic sections at day 7 after start of DSS administration. Right panel: HCS at day 7. (e) SPF C57BL/6 WT mice and SPF *Tlr5*^−/−^ mice aged 6 to 8 weeks were administered 3.5% DSS in drinking water at day 0. Mice were additionally treated with EcN (DSS + EcN) or an EcNΔ*fliC* deletion mutant (DSS + EcNΔ*fliC*) resuspended in DSS-containing drinking water at 10^8^ bacteria mL^−1^. Left panel: HE-stained colonic sections at day 7 after start of DSS administration. Right panel: HCS at day 7. Statistics: (a), (b), (c), (e), one-way ANOVA with Tukey multiple comparison test; (d) Kruskal–Wallis test with multiple comparisons. *p*-values < 0.05 are considered to represent statistical significance. (a–e) The data underlying this figure can be found in S1 Data. DSS, dextran sodium sulphate; EcN, *E*. *coli* Nissle 1917; *fliC*, flagellin; HCS, histological colitis score; HE, hematoxylin–eosin; MG1655, *E*. *coli* K12 MG1655; MPK, *E*. *coli* mpk; SPF, specific-pathogen–free; TLR, Toll-like receptor; WT, wild type.

EcN is the only symbiont with verified symbiotic effects on the outcome and progression of UC in humans [8] as well as in DSS-induced colitis in mice [38–40]. For these reasons, we decided to use the DSS-induced colitis model for the elucidation of *E*. *coli*-mediated effects on colitis pathology. Administration of 3.5% DSS in the drinking water led to strong loss of body weight in wild-type (WT) C57BL/6 mice (Fig 1A), accompanied by severe tissue changes and leukocyte influx, as indicated by high histological colitis scores (HCSs) (Fig 1B). In agreement with previous studies [38–40], administration of viable symbiotic EcN provided beneficial effects on the outcome of DSS-induced colitis in WT BL/6 mice, characterized by drastically reduced weight loss and HCS (Fig 1A and 1B). Therefore, we were interested in how other commensal *E*. *coli* strains influence the progress and outcome of DSS-induced colitis and which structural features of these strains might mediate potentially observed differences.

Therefore, we administered two additional *E*. *coli* strains in the same concentration as EcN, which resulted in comparable *E*. *coli* colony-forming units (CFUs) in the feces of all groups (S1 Fig), and monitored disease progression and outcome compared to DSS-only–treated WT mice; specifically, the well-known commensal strains (1) *E*. *coli* K12 MG1655 (MG1655) and (2) *E*. *coli* mpk (MPK) [41–44] were used. Importantly, these strains do not mediate symbiotic properties in various mouse models for microbiota-influenced pathologies [41,43,45], and in contrast to treatment with EcN, administration of MPK and MG1655 did not provide any beneficial effects on the outcome of DSS-induced colitis since the HCS of both groups was comparable to the DSS-only control group (Fig 1B), and weight loss was only slightly reduced.

Given the direct effect of flagellin on TLR5-signaling and its impact on intestinal immune homeostasis [36,37], we hypothesized that the protective effect of EcN was at least partially mediated by its flagella. In order to test this hypothesis, an EcN mutant lacking the *fliC* gene encoding for flagellin (EcNΔ*fliC*) [46] was administered to DSS-treated mice. While EcN, MG1655, and MPK each harbor a functional flagellum (S2 Fig), EcNΔ*fliC* does not (S3 Fig). Fecal *E*. *coli* CFUs in all bacteria-treated groups were comparable (S1A Fig). Whereas weight loss was only partially rescued by administration of live EcNΔ*fliC* in the drinking water (Fig 1C), absence of flagellin in this strain completely abrogated the positive effect on histological damage observed for flagellin-expressing EcN and was comparable to DSS-only–treated control mice, providing strong signs of intestinal inflammation (Fig 1D). This indicated that the presence of flagella is crucial for mediation of protective properties of EcN in this mouse model. Since the flagellum and its constituent protein flagellin are recognized by host TLR5, we performed DSS-induced colitis experiments using TLR5-deficient (*Tlr5*^−/−^) mice.

Dependent on the housing conditions, *Tlr5*^−/−^ mice either spontaneously develop a chronic form of colitis and/or metabolic syndrome [37,47,48] or not [49]. Our specific-pathogen–free (SPF) housing conditions did not lead to spontaneous intestinal disorders and did not differently affect the outcome of DSS-induced colitis in *Tlr5*^−/−^ mice compared to their equally treated WT counterparts (Fig 1E), though *Tlr5*^−/−^ mice treated with EcN showed significantly increased histological damage compared to EcN-treated WT mice, resembling the disease phenotype that was observed in DSS-only–treated WT and *Tlr5*^−/−^ mice (Fig 1E). Administration of EcNΔ*fliC* to DSS-treated *Tlr5*^−/−^ mice did not positively affect the outcome of experimental colitis, as demonstrated by HCSs comparable to EcNΔ*fliC*-treated WT mice and to DSS-only–treated mice of both genotypes (Fig 1E; see also S1 Table for detailed statistical analysis). Importantly, fecal *E*. *coli* CFUs in all bacteria-treated groups in *Tlr5*^−/−^ mice were comparable (S1B Fig). Therefore, we concluded that the beneficial effects of symbiotic EcN on the progress and outcome of DSS-induced colitis is mainly mediated by the interaction of host TLR5 with EcN flagella.

### Flagella-mediated inflammation-suppressing effects are specific for EcN flagella-enriched preparations and are not mediated by preparations from other commensal *E*. *coli* strains

**Fig 2:**
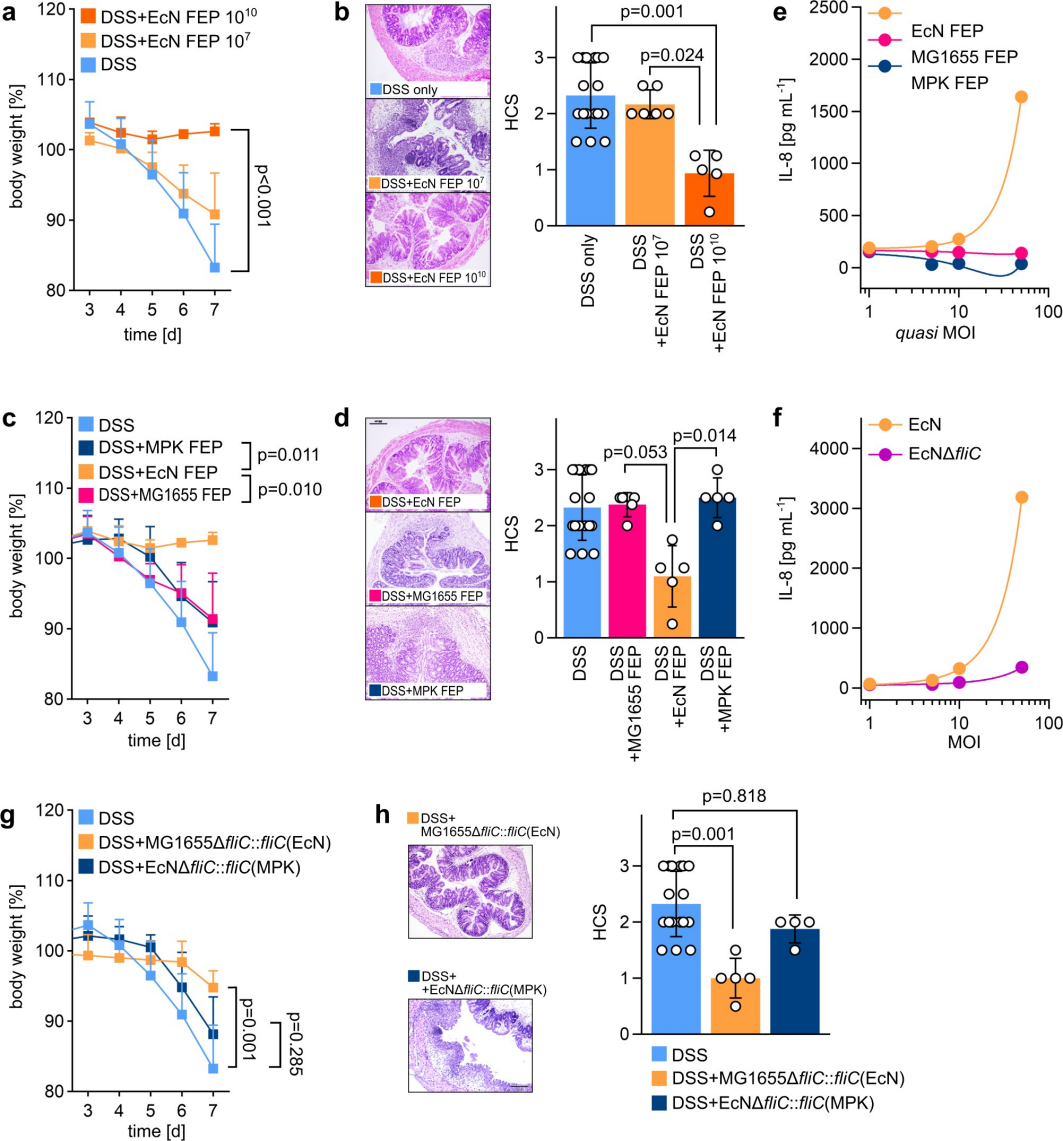
Flagella-dependent influence of different commensal *E*. *coli* strains on DSS-induced colitis in WT mice. (a + b) SPF C57BL/6 WT mice aged 6 to 8 weeks were administered 3.5% DSS in drinking water at day 0. Mice were additionally treated with FEPs obtained from 10^7^ (EcN FEP 10^7^) and 10^10^ (EcN FEP 10^10^) EcN per 100 mL drinking water. (a) Change in body weight relative to start of DSS administration at day 0. (b) Left panel: HE-stained colonic sections at day 7 after start of DSS administration. Right panel: HCS at day 7. (c + d) SPF C57BL/6 WT mice aged 6 to 8 weeks were administered 3.5% DSS in drinking water at day 0. Mice were additionally treated with FEPs obtained from 10^10^ EcN, MG1655, or MPK per 100 mL drinking water. (c) Change in body weight relative to start of DSS administration at day 0. (d) Left panel: HE-stained colonic sections at day 7 after start of DSS administration. Right panel: HCS at day 7. (e) mTLR5-HEK293 cells were stimulated with FEP obtained from EcN, MG1655, and MPK for 24 h. FEPs were generated from the number of bacteria corresponding to a certain MOI (quasi-MOI). Resulting IL-8 secretion into cell supernatant as a result of TLR5 receptor activation was detected by ELISA. (f) mTLR5-HEK293 cells were stimulated with EcN and EcNΔ*fliC* at different MOI for 24 h. Resulting IL-8 secretion into cell supernatant as a result of TLR5 receptor activation was detected by ELISA. (g + h) SPF C57BL/6 WT mice aged 6 to 8 weeks were administered 3.5% DSS in drinking water at day 0. Mice were additionally treated with 10^10^ viable bacteria of the indicated complementation mutants per 100 mL DSS-containing drinking water. (g) Change in body weight relative to start of DSS administration at day 0. (h) Left panel: HE-stained colonic sections at day 7 after start of DSS administration. Right panel: HCS at day 7. Statistics: (a), (c), (g), one-way ANOVA with Tukey multiple comparison test; (b), (d), (h), Kruskal–Wallis test with multiple comparisons. *p*-values < 0.05 are considered to represent statistical significance. The data underlying this figure can be found in S1 Data. DSS, dextran sodium sulphate; EcN, *E*. *coli* Nissle 1917; FEP, flagella-enriched preparation; *fliC*, flagellin; HCS, histological colitis score; HE, hematoxylin–eosin;; IL, interleukin; MG1655, *E*. *coli* K12 MG1655; MOI, multiplicity of infection; MPK, *E*. *coli* mpk; mTLR5-HEK293 cell, mouse-TLR5–expressing human embryonic kidney 293 cell; SPF, specific-pathogen–free; TLR, Toll-like receptor; WT, wild type.

Since all three *E*. *coli* strains used—EcN, MPK, and MG1655—expressed a functional flagellum, we hypothesized that the different effects on DSS-induced colitis mediated by distinct *E*. *coli* strains were not caused by the presence of a flagellum per se but rather were rooted in structural differences in their flagellin protein structures. To test this and to exclude the contribution of factors requiring viable bacteria, we generated flagella-enriched preparations (FEPs) from symbiotic EcN and nonsymbiotic MG1655 and MPK as described in the experimental procedures, and these FEPs were devoid of any viable bacteria. Fig 2A demonstrates that the EcN FEP recapitulated the protective effects on the outcome of DSS-induced colitis in WT mice observed for viable EcN. This effect was dose dependent, with the FEP obtained from 10^10^ EcN per mL drinking water providing the strongest inflammation-reducing effects, as indicated by absent weight loss (Fig 2A) and low HCS of DSS-treated animals (Fig 2B).

According to the results shown in Fig 2A and 2B, we performed all further experiments by default with the FEP obtained from 10^10^ bacteria per mL drinking water, which was thought to induce a strong TLR5-mediating signaling. Next, we compared the FEP from EcN with the FEPs obtained from MPK (MPK FEP) and MG1655 (MG1655 FEP). We verified that FEPs generated from each of the tested strains contained similar concentrations of endotoxin, overall protein, and flagellin (S4A–S4E Fig). Thus, DSS-treated WT mice were administered MPK FEP as well as MG1655 FEP and compared to DSS-treated WT mice, which were administered EcN FEP. As demonstrated in Fig 2C and 2D, neither MPK FEP nor MG1655 FEP provided any beneficial effects concerning DSS-induced colitis in WT mice, indicating that the molecular nature of flagellin impacted on this process.

To assess whether this was reflected at the level of TLR5 activation, we used human embryonic kidney cells overexpressing mouse TLR5 (mTLR5-HEK293), stimulated them with either viable bacteria or FEPs, and measured the resulting interleukin (IL)-8 secretion to quantify TLR5-dependent NFκB activation. When comparing EcN FEP, MG1655 FEP, and MPK FEP, the latter FEPs from nonsymbiotic bacteria completely failed to activate the mouse TLR5 receptor in any tested concentration, while EcN FEP provided a concentration-dependent signaling intensity, as demonstrated by differential NFκB-activation–dependent IL-8 secretion from mTLR5-HEK293 cells (Fig 2E). Of note, a strong effect on NFκB activation was also observed for viable EcN but not EcNΔ*fliC* (Fig 2F). Thus, we assumed that flagellin of symbiotic EcN might feature a distinct property that is absent in nonsymbiotic *E*. *coli* strains. To explore this in detail, different complementation mutants were generated: (1) an MG1655Δ*fliC* mutant was complemented with the *fliC* gene from EcN (MG1655Δ*fliC*::*fliC*(EcN)), and (2) an EcNΔf*liC* mutant was complemented with the *fliC* gene from MPK (EcNΔ*fliC*::*fliC*(MPK)) (see S5 Fig for details on complementation). Both mutants provided efficient flagella expression and adequate mobility as demonstrated by electron microscopy and bacterial swarming assays (S6 Fig), indicating that the exchange mutation did not negatively affect flagellum expression and function. Both complemented viable strains were administered to DSS-treated mice, and the disease outcome was monitored as before (Fig 2G and 2H). Although a slight reduction in body weight was observed in DSS-treated animals that were administered MG1655Δ*fliC*::*fliC*(EcN) (Fig 2G), the damage of colonic tissue was significantly lower compared to DSS-treated mice without additional bacterial administration (Fig 2H). However, administration of EcNΔ*fliC*::*fliC*(MPK) did not provide any beneficial effects on DSS-induced colitis, as indicated by strong weight loss and increased HCS (Fig 2G and 2H). Therefore, we concluded that the inflammation-reducing features of the EcN flagella are specific for this strain and are not present in the flagella of MG1655 or MPK. However, this raises the question of which structural features might account for the differences of the EcN FliC protein compared to FliC from nonsymbiotic *E*. *coli* strains.

### Insertions within the HVR of flagellin account for the symbiotic effects of EcN compared to nonsymbiotic *E*. *coli* strains

**Fig 3:**
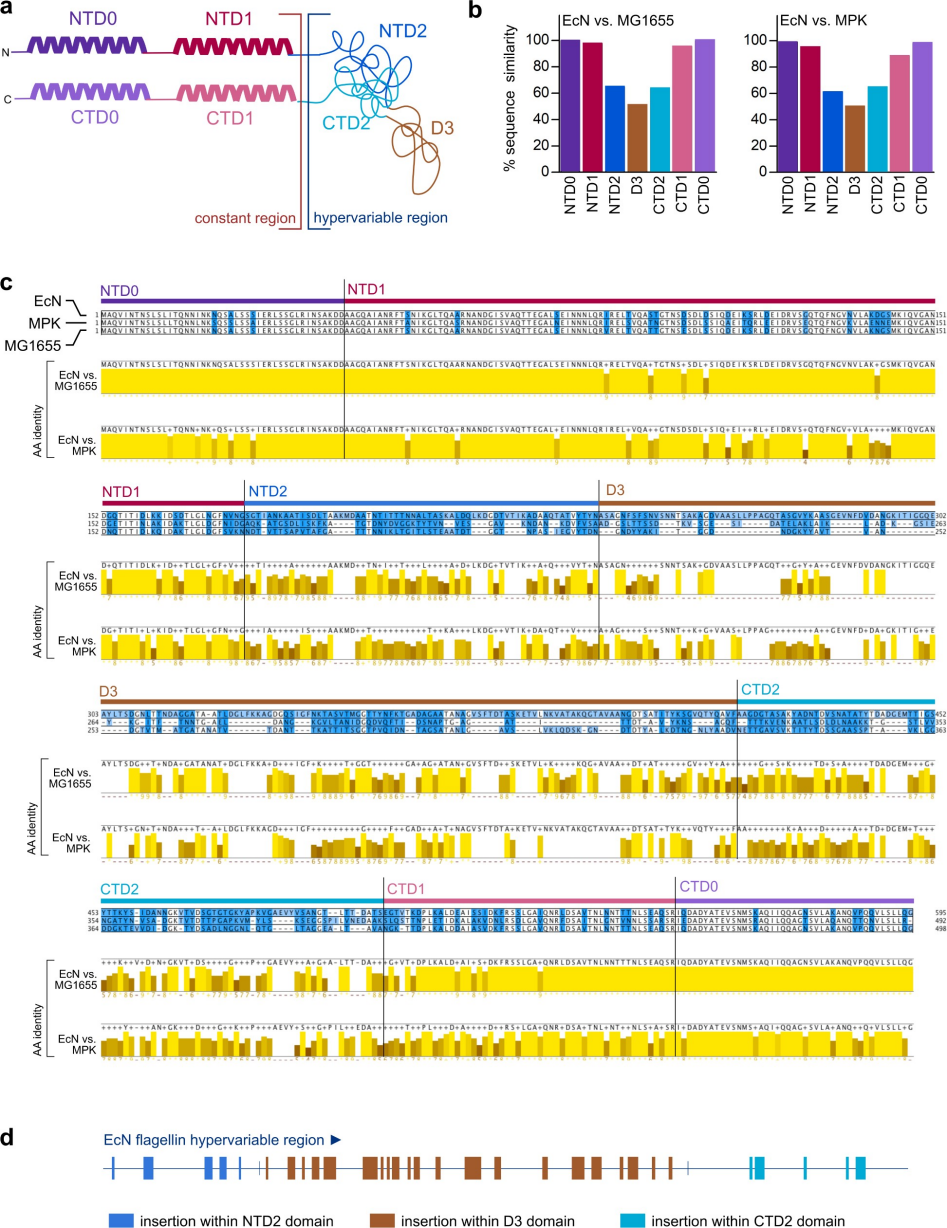
Detailed comparison of the flagellin amino-acid sequences of different *E*. *coli* strains. Protein alignment of FliC proteins from EcN (CCQ05465.1), MPK, and MG1655 (NP_416433.1) genomes. (a) Schematic structure of flagellin according to Yonekura and colleagues [50,51]. D0 and D1 comprise conserved N- and C-termini of *fliC*, packed into α-helical structures in the filament core. The NTD2/CTD2- and D3-domain–containing HVR is attached adjacent to the D2 domains, located at the outer surface of the filament. (b) Quantification of amino-acid sequence similarities of all 6 flagellin domains. Computation was performed using similarity indices depicted in 3c. (c) The alignment was generated using MAFFT. Amino acids were colored by overall conservation (white: fully conserved, light blue to dark blue: high to low conservation). Consensus sequence and sequence conservation for pairwise comparisons of EcN to MPK and MG1655, respectively, are shown in yellow. Darker shades of yellow represent lower sequence conservation. (d) Schematic overview of the differences of the EcN flagellin HVR compared to the HVRs of both MG1655 and MPK. Sequences (insertions) that are only present in EcN HVR and not in MPK HVR or MG1655 HVR are highlighted as colored squares. AA, amino acid; CTD, C-terminal domain; EcN, *E*. *coli* Nissle 1917; *fliC*, flagellin; HVR, hypervariable region; MAFFT, Multiple Alignment using Fast Fourier Transform; MG1655, *E*. *coli* K12 MG1655; MPK, *E*. *coli* mpk; NTD, N-terminal domain.

FliC proteins consist of a constant region comprising one N- and one C-terminal D0 and D1 domain (NTD0, NTD1, CTD0, and CTD1). Some bacteria—e.g., *Escherichia* and *Salmonella* strains—additionally contain an HVR composed of a C- and N-terminal D2 (NTD2, CTD2) domain as well as a central D3 domain (Fig 3A). As expected, sequence alignment comparisons between FliC of EcN, MG1655, and MPK revealed that in the constant region, the sequence similarity of the 4 domains exceeds 95% (Fig 3B and 3C). This constant region is thought to be the primary mediator of TLR5 activation [30,28], However, sequence similarity of the N- and C-terminal D2 domains within the HVR is 65% (NTD2) and 64% (CTD2) between EcN FliC and MG1655 FliC, as well as 61% (NTD2) and 64% (CTD2) in the case of comparing EcN FliC with MPK FliC. The differences between D3 domain sequences is even lower than 50% sequence similarity for both comparisons (Fig 3B and 3C). These low numbers in relative sequence similarity are mainly due to the presence of numerous sets of amino-acid inserts within the HVR of EcN flagellin compared to the corresponding regions of MG1655 flagellin and MPK flagellin (Fig 3D), thus rendering the NTD2, D3, and CTD3 domains of EcN substantially longer. However, not only the length but also the amino-acid sequence of the HVR is highly different, both in comparison between EcN and MG1655 or EcN and MPK and also between MG1655 and MPK.

### Deletions within the HVR of FliC leads to loss of EcN inflammation-reducing properties

**Fig 4:**
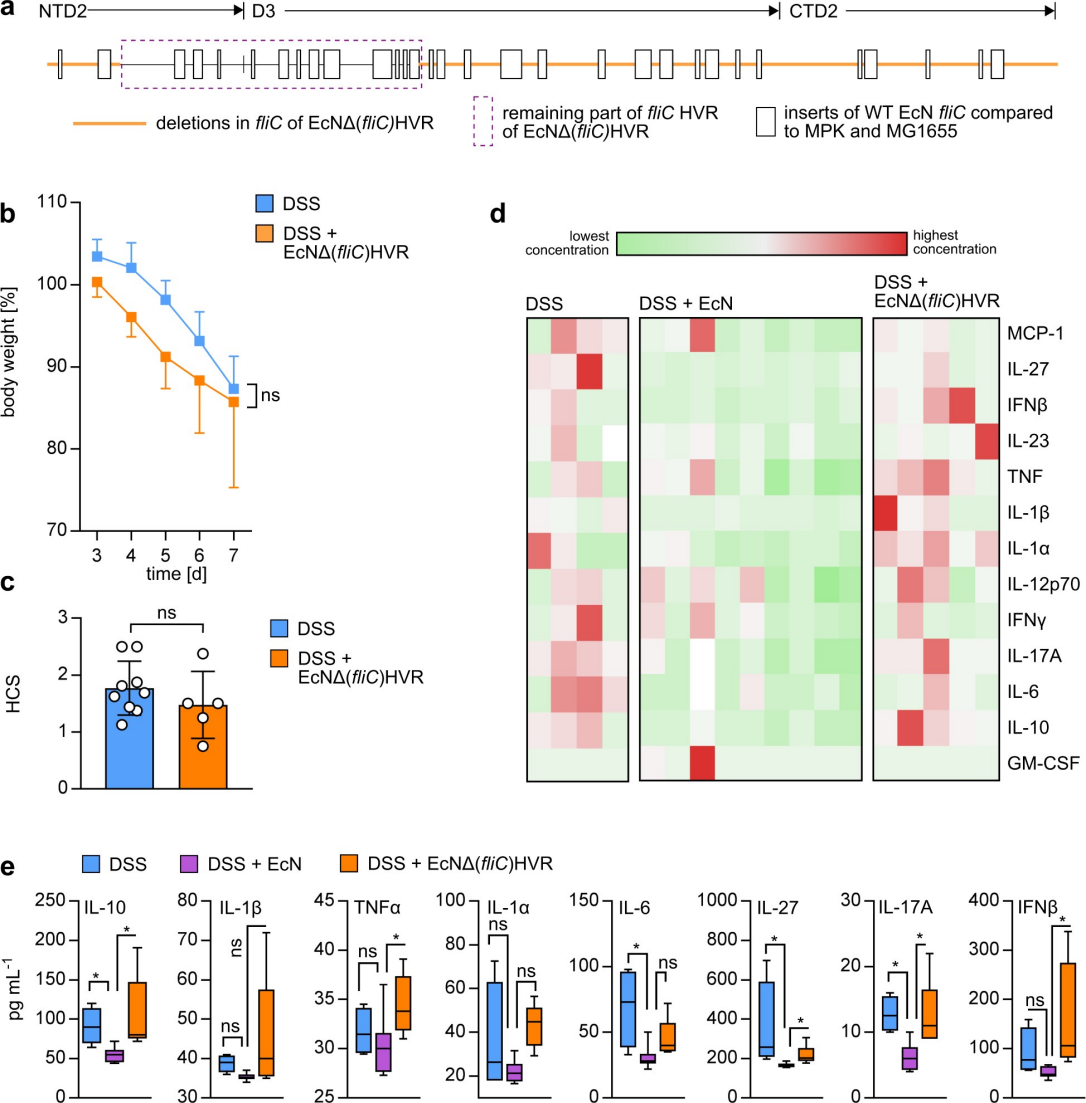
Partial deletion of the EcN HVR leads to loss of EcN probiotic effects. (a) Schematic display of the deleted parts in the EcNΔ(*fliC*)HVR mutant. The orange line depicts the parts of the EcN HVR that were deleted. (b + c) SPF C57BL/6 WT mice aged 6 to 8 weeks were administered 3.5% DSS in drinking water at day 0. Mice were additionally treated with 10^10^ viable bacteria of EcN or the EcNΔ(*fliC*)HVR mutant resuspended in 100 mL DSS-containing drinking water. (b) Change in body weight relative to start of DSS administration at day 0. (c) HCS at day 7. Statistical analysis was performed using Student *t* test. Error bars represent SD. White dots in column bars represent each biological replicate. (d) Heat map of cytokine concentrations in serum from DSS-treated mice. Each column represents a different individual. (e) Detailed analysis of cytokine concentrations from serum depicted in (d). Asterisks indicate statistical significance. All shown cytokines provide the strongest differences between DSS-treated and DSS + EcNΔ*fliC*(HVR)-treated as well as between DSS + EcN and DSS + EcNΔ*fliC*(HVR)-treated groups. See S1 Table for detailed statistical analysis using Kruskal–Wallis test with multiple comparisons. *p*-values < 0.05 are considered to represent statistical significance. (b + c + e) The data underlying this figure can be found in S1 Data. CTD, C-terminal domain; DSS, dextran sodium sulphate; EcN, *E*. *coli* Nissle 1917; *fliC*, flagellin; GM-CSF, granulocyte-macrophage colony-stimulating factor; HCS, histological colitis score; HVR, hypervariable region; IFN, interferon; IL, interleukin; MCP, monocyte chemoattractant protein; MG1655, *E*. *coli* K12 MG1655; MPK, *E*. *coli* mpk; NTD, N-terminal domain; ns, not significant; SPF, specific-pathogen–free; TNF, tumor necrosis factor; WT, wild type.

In order to check whether the different HVR structure is causative for the observed disparities in influencing the progress of DSS-induced colitis, we deleted large parts of the EcN *fliC* HVR, namely a short part of the NTD2 domain as well as large parts of D3 and the entire CTD2 domain (EcNΔ*fliC*(HVR)) (Fig 4A). This deletion resulted in a significantly shorter assembled flagellum compared to WT EcN, providing only about 10% of the length of WT EcN flagella (S7 Fig). We administered EcNΔ*fliC*(HVR) as a viable bacterium to DSS-treated mice and checked for changes in body weight, followed by histological analysis of the colonic tissue 7 days after the start of DSS treatment. As demonstrated in Fig 4B and 4C, there were no detectable inflammation-preventing effects when the EcNΔ*fliC*(HVR) deletion mutant was used, as indicated by strong weight loss (Fig 4B) and high HCSs (Fig 4C), which was in sharp contrast to WT EcN (Fig 1A–1D). In conclusion, these experiments show that insertions within the flagellin HVR are responsible for the disease-ameliorating properties of EcN flagellin. To gain insight into the effect of HVR modulation on systemic inflammation, we determined serum concentrations of 13 pro- and anti-inflammatory cytokines in DSS + EcN-treated, DSS + EcNΔ*fliC*(HVR)-treated, and DSS-only–treated mice to characterize the influence of the flagellin HVR on the cytokine-associated protective properties of EcN in this mouse model. Consistent with reports showing that DSS-treated mice provide increased serum levels of, e.g., tumor necrosis factor (TNF)α, IL-6, IL-1β, and IL-17 compared to healthy non-DSS–treated controls [52,53], colonic inflammation led to a systemic increase of proinflammatory cytokines, which translated into elevated serum concentrations. Fig 4E depicts a heat map of all detected cytokines in each mouse of the three respective groups. We detected significantly higher serum levels of the proinflammatory cytokines IL-1β, IL-1α, TNFα, IL-6, and IL-17A as well as of the anti-inflammatory cytokines IL-27, IL-10, and interferon (IFN)β in DSS-only–and DSS + EcNΔfliC(HVR)-treated mice compared to DSS + EcN-treated mice (Fig 4E, see also S1 Table for detailed statistical analysis). Therefore, we concluded that the presence of the full HVR is a precondition for the EcN-mediated symbiotic effects during DSS-induced colitis in WT mice, and its deletion results in loss of protection against intestinal inflammation.

### EcN-flagellin–induced protective effects are mediated by host TLR5^+^CD11c^+^ cells in the colonic LP

**Fig 5:**
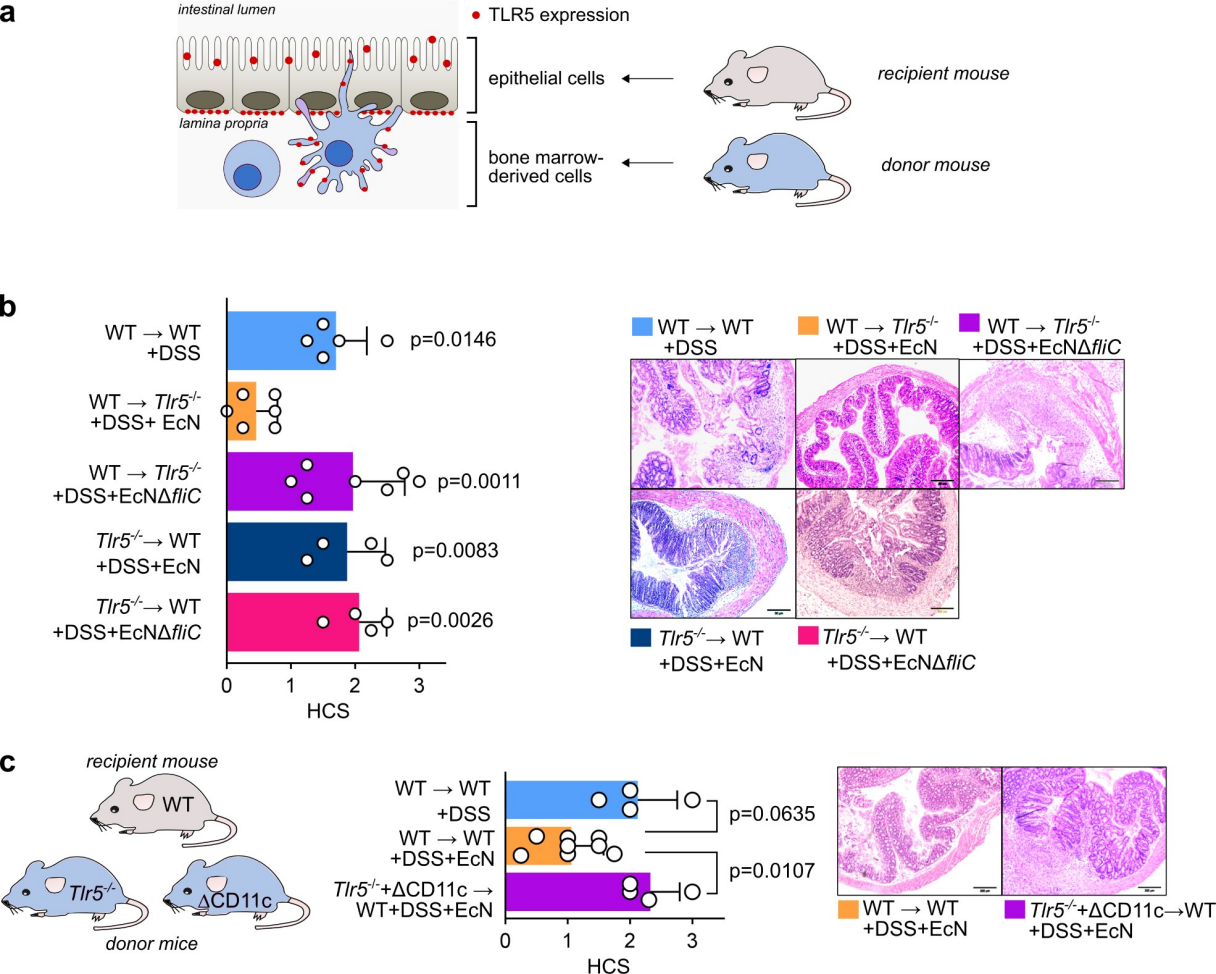
EcN-flagellin–induced protective effects are mediated by host TLR5^+^CD11c^+^ cells in the cLP. (a) Schematic depiction of the generation of BMCM as described in the experimental procedures. Red dots represent the typical sites of TLR5 expression in the mouse intestine. (b) Different SPF BMCM were administered 3.5% DSS in drinking water at day 0. Mice were additionally treated with 10^10^ viable bacteria of EcN or the EcNΔ(*fliC*)HVR mutant resuspended in 100 mL DSS-containing drinking water. See text for further information on the nomenclature of distinct BMCM groups. Indicated *p*-values refer to the comparison of the respective data set with the WT → *Tlr5*^−/−^ + DSS + EcN group. Upper panel: HCS at day 7. Lower panel: HE-stained colonic sections at day 7 after start of DSS administration. (d) Irradiated WT recipient mice were transplanted with bone marrow from *Tlr5*^−/−^ and ΔCD11c donor mice in a 1:1 ratio. Mice were administered 3.5% DSS in drinking water at day 0. Mice were additionally treated with 10^10^ viable bacteria of EcN resuspended in 100 mL DSS-containing drinking water and compared to control groups. See text for further information on the BMCM groups nomenclature. Middle panel: HCS at day 7. Right panel: HE-stained colonic sections at day 7 after start of DSS administration. Statistics: (b) One-way ANOVA with Tukey multiple comparison test, (c) Mann–Whitney test, (d) Kruskal–Wallis test with multiple comparisons, *p*-values < 0.05 are considered to represent statistical significance. (b + c) The data underlying this figure can be found in S1 Data. BMCM, bone-marrow–chimeric mice; cLP, colonic LP; DSS, dextran sodium sulphate; EcN, *E*. *coli* Nissle 1917; *fliC*, flagellin; HCS, histological colitis score; HE, hematoxylin–eosin; HVR, hypervariable region; LP, lamina propria; SPF, specific-pathogen–free; TLR, Toll-like receptor; WT, wild type.

So far, we have demonstrated that symbiotic EcN mediated at least a remarkable part of its symbiotic properties through its flagellin HVR. This effect was dependent on TLR5 expression in the host. Therefore, we wanted to further elucidate which TLR5-expressing host cell population was pivotal for the mediation of the EcN-flagellin–induced effects. The crucial contribution of TLR5 expression on intestinal epithelial cells (IECs) for maintenance of intestinal homeostasis has already been reported [37]. Colonic IECs do not only express TLR5 on their basolateral side [54]. A recent publication demonstrated that colonic IECs also express TLR5 on the luminal side, thus secreting antimicrobial peptides and cytokines in response to flagellin recognition [55]. However, TLR5-expressing intestinal LP dendritic cells (DCs) also contribute to flagellin-mediated immune responses [37,56]. Therefore, we were interested in which intestinal cell population mediated the observed symbiotic flagellin-induced inflammation-silencing properties. Since IECs belong to the group of stromal cells and antigen-presenting cells such as DCs are derived from hematopoietic stem cells, one can investigate the distinct influence of these two differentially originated cell types by using bone-marrow–chimeric mice (BMCM) (Fig 5A). Therefore, we generated different groups of BMCM: (1) WT BL/6 recipient mice transplanted with bone marrow from *Tlr5*^−/−^ donor mice (*Tlr5*^−/−^ → WT), (2) *Tlr5*^−/−^ recipient mice transplanted with bone marrow from WT BL/6 donor mice (WT → *Tlr5*^−/−^), and (3) as controls, WT BL/6 recipient mice transplanted with bone marrow from WT BL/6 donor mice (WT → WT). All these animals were SPF-housed and treated with DSS and EcN, DSS and EcNΔ*fliC*, or DSS only. As demonstrated in Fig 5B, WT → *Tlr5*^−/−^ mice that were administered EcN provided low HCS, indicating low colonic inflammation, while the protective effect of EcN was completely abolished using *Tlr5*^−/−^ → WT mice (Fig 5B). As seen in WT BL/6 mice (Fig 1), both groups of BMCM developed severe colitis symptoms when administered DSS and the EcNΔ*fliC* deletion mutant. Therefore, we concluded that the protective effect of EcN flagellin is mainly mediated by cells of hematopoietic origin. CD11c^+^ cells such as DCs are one major cell type from the hematopoietic cell lineage mediating microbiota-derived anti-inflammatory processes in the intestine [41,57]. Therefore, we had a closer look at CD11c^+^ cells in the colonic LP (cLP) and their relevance for EcN-mediated symbiotic effects during DSS-induced colitis.

We generated BMCM restricting TLR5 deficiency largely to CD11c^+^ cells with a substantial amount of other hematopoietic cells still expressing TLR5 (*Tlr5*^−/−^ + ΔCD11c → WT). By using *Tlr5*^−/−^ and ΔCD11c donor mice for bone marrow transplantation, we ensured that at least half of hematopoietic cells other than CD11c^+^ cells do express TLR5. Generation of these BMCM was strictly validated (S8 Fig). As demonstrated in Fig 5C, this group provided significantly higher inflammation when treated with DSS and EcN compared to WT → WT control animals that were treated equally. This finding strongly supports the idea that TLR5^+^CD11c^+^ cells in the cLP are the main mediators of the symbiotic effects caused by EcN flagellin.

### Administration of symbiotic recombinant flagellin prevents intestinal inflammation

**Fig 6:**
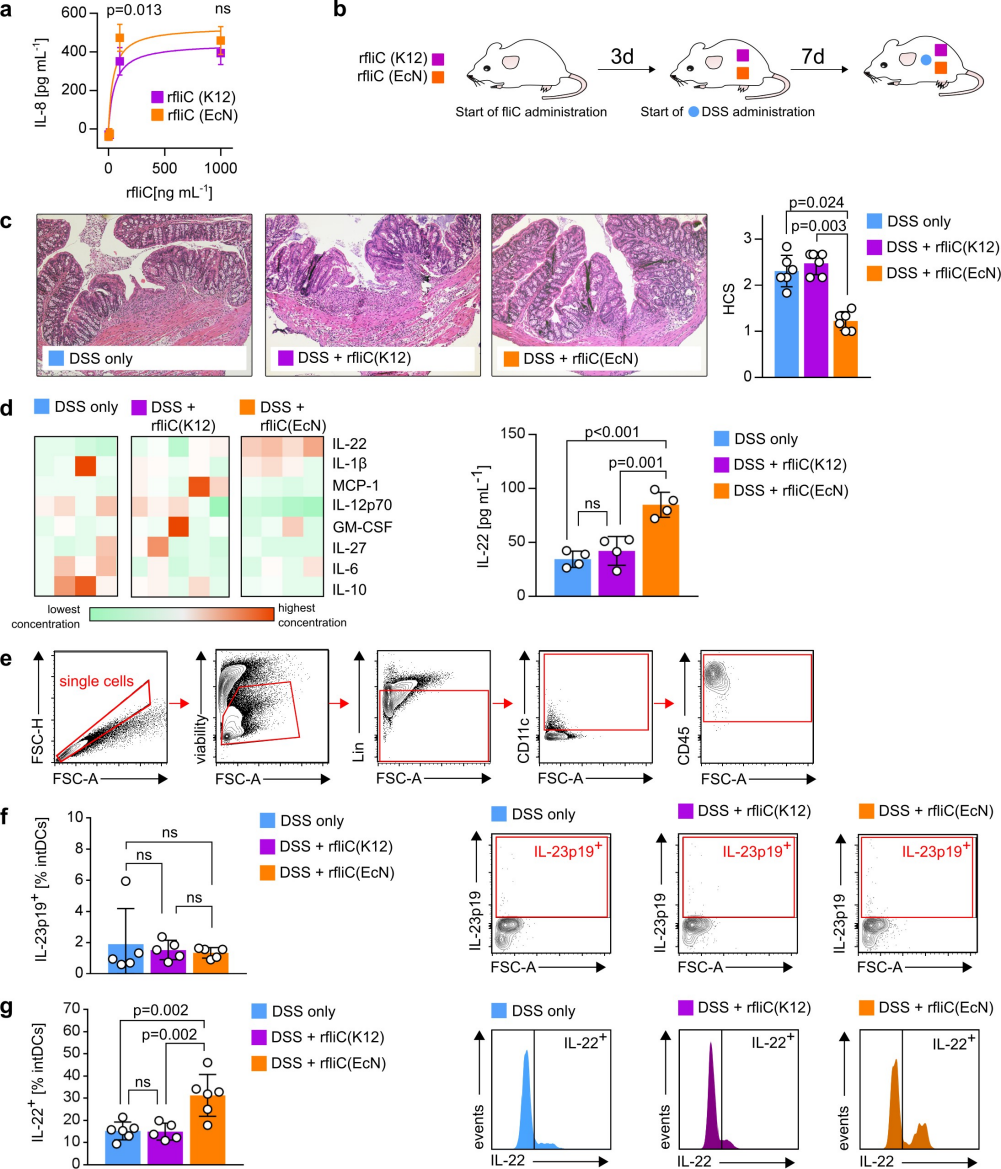
Recombinant FliC protects against DSS-induced colitis. (a) mTLR5-HEK293 cells were stimulated with rfliC(K12) or rfliC(EcN) for 24 h. Resulting IL-8 secretion into cell supernatant as a result of TLR5 receptor activation was detected by ELISA. (b) Experimental setup: SPF C57BL/6 WT mice aged 6 to 8 weeks were administered 2 μg recombinant flagellin daily via intragastral gavage. 3 days after start of flagellin administration, 3.5% DSS was added to the drinking water. Progress of DSS-induced colitis was monitored for additional 7 days. (c) HCS and representative HE-stained colonic sections at day 7. Statistical analysis was performed using the Kruskal–Wallis test. Error bars represent SD. White dots in column bars represent each biological replicate. (d) Heat map of cytokine concentrations in serum from DSS-treated mice as shown in (b). Each column represents a different individual. Right panel: IL-22 concentration in blood serum. Statistical analysis was performed using one-way ANOVA. Error bars represent SD. White dots in column bars represent each biological replicate. (e) Gating strategy to define the population of intDCs from the cLP. Lin = Ly6G/C, CD45R, CD64. (f + g) Proportion of IL-23^+^ (f) or IL-22^+^ (g) intDCs from the experiment shown in (b). Representative histograms or contour blots are shown. Statistical analysis was performed using one-way ANOVA. Error bars represent SD. White dots in column bars represent each biological replicate. *p*-values < 0.05 are considered to represent statistical significance. (a + c + d + f + g) The data underlying this figure can be found in S1 Data. cLP, colonic LP; DC, dendritic cell; DSS, dextran sodium sulphate; EcN, *E*. *coli* Nissle 1917; *fliC*, flagellin; FSC, forward scatter; HCS, histological colitis score; HE, hematoxylin–eosin; IL, interleukin; intDC, intestinal DC; LP, lamina propria; MG1655, *E*. *coli* K12 MG1655; mTLR5-HEK293 cell, mouse-TLR5–overexpressing human embryonic kidney 293 cell; ns, not significant; rfliC(EcN), recombinant flagellin from EcN; rfliC(K12), recombinant flagellin from MG1655; SPF, specific-pathogen–free; TLR, Toll-like receptor.

According to our results, we proposed flagellin from a symbiotic *E*. *coli* strain to be a suitable agent in order to prevent pathological intestinal inflammation that was supposed to be rooted in its increased TLR5 activation capacity. To finally provide evidence for this hypothesis, we aimed to administer recombinant flagellin from symbiotic and nonsymbiotic *E*. *coli* strains to DSS-treated WT mice. Therefore, we used recombinant flagellin from symbiotic EcN (rfliC(EcN)) and from nonsymbiotic MG1655 (rfliC(K12)). While rfliC(K12) was commercially available, rfliC(EcN) was generated and quality-checked as described in the supplementary material (S9 and S10 Figs). First, we checked for their ability to induce TLR5 receptor activation. As seen in Fig 6A, both recombinant flagellins induced IL-8 secretion in mTLR5-HEK293 cells in a concentration-dependent manner. However, rfliC(EcN) induced higher IL-8 secretion compared to rfliC(K12) at a medium-range concentration of 100 ng mL^−1^, thus indicating stronger TLR5 receptor activation. Next, we aimed to test the inflammation-preventing properties of both recombinant flagellins during DSS-induced colitis in mice. Therefore, we used WT SPF C57BL/6 mice and started daily intragastral gavage of 2 μg recombinant flagellin 3 days prior to start of DSS administration. DSS was administered as 3.5% solution in drinking water, and flagellin administration was continued daily until the end of the experiment, 7 days after initial DSS exposure (Fig 6B). The non-flagellin–administered DSS-only–treated control group was gavaged daily with 100 μL sterile PBS, the solvent flagellin was reconstituted in. As demonstrated in Fig 6C, administration of rfliC(EcN) resulted in significantly lower tissue damage in the colon compared to rfliC(K12)-treated and DSS-only–treated mice, therefore indicating prevention of intestinal inflammation. Lower HCSs in rfliC(EcN)-treated mice were associated with decreased concentrations of various proinflammatory cytokines in the blood serum. However, we detected increased serum levels of IL-22 in rfliC(EcN)-treated mice compared to both other groups (Fig 6D). Since IL-22 contributes to maintenance of intestinal immune homeostasis by strengthening the intestinal epithelial barrier, we assumed that EcN-flagellin–induced stronger TLR5-activation was associated with increased IL-22 expression. The main sources of intestinal IL-22 are reported to be innate lymphoid cells (ILC3s) and CD4^+^ T cells, which secrete IL-22 in response to IL-23 [58,59]. Since CD11c is expressed on various different cell types in the mouse intestine, we further focused on intestinal DCs. Intestinal DCs (intDCs) were defined as being Ly6G/Ly6C^neg^, CD45R/B220^neg^, CD64^neg^, CD45^pos^, and CD11c^pos^ (Fig 6E). Focusing on intDCs in the cLP, however, did not reveal any differences in IL-23 expression between all compared groups (Fig 6F). This prompted us to check whether intDCs themselves might be the source of IL-22. In fact, we detected significantly increased proportions of IL-22^+^ cells in rfliC(EcN)-treated mice compared to both other groups (Fig 6F), which correlated with a lower HCS (Fig 6C). Therefore, we assumed that enhanced IL-22 expression by these cells contributed to symbiotic-flagellin–mediated intestinal immune homeostasis maintenance in DSS-induced colitis.

## Discussion

*E*. *coli* represents one of the most intensely studied intestinal commensals. Colonization of the human gut with *E*. *coli* starts early after birth even before colonization with anaerobes, which usually make up the largest part of the human intestinal microbiota [60]. However, distinct commensal *E*. *coli* strains can provide completely different immunogenic properties in a certain host’s intestine. While pathobiotic commensal *E*. *coli* strains may mediate pathophysiological immunological processes in a predisposed host, symbiotic commensal *E*. *coli* strains may exhibit even strong probiotic features. Symbionts with beneficial features are widely used to restore gut homeostasis in human patients and animal models [61]. One of the most prominent and therefore most extensively studied symbiotic *E*. *coli* strains is EcN. However, its symbiotic properties have not yet been linked to a certain molecular or structural feature. Interestingly, studies using outer-membrane vesicles from EcN have revealed that these vesicles are able to mimic the properties of viable EcN, thus indicating that any surface-associated component might be crucial for the mediation of its symbiotic capacities [62]. An important surface-associated molecule of EcN is flagellin, the constitutive protein of the bacterial flagellum. Most clinically relevant *E*. *coli* strains express flagella on the cell surface [63], thus rendering bacteria motile and facilitating bacterial attachment to the intestinal mucus [46]. Since flagellin represents one of the most important antigens in IBD patients [33], we assumed that the definite flagellin structure might contribute to the symbiotic properties of certain *E*. *coli* strains and is therefore involved in modulation of IBD pathogenesis.

With this study, we found the HVR of flagellin to strongly influence the immunogenic properties of the tested commensal *E*. *coli* strains. Unexpectedly, we observed that symbiotic EcN harbors a substantially longer HVR, comprised of various small inserts, compared to nonsymbiotic commensal *E*. *coli* strains. Previous work on structure-function relationship for flagellin–TLR5 interactions have focused on the so-called N- and C-terminal D0 and D1 domains of flagellin that make up the protein’s constant region and that are highly conserved among all flagellins across different bacterial phyla [50,51]. This constant region was also characterized as being crucial for interaction with TLR5 and therefore for TLR5-mediated intracellular signaling in host cells [29,30]. Given the high similarity of EcN, MPK, and MG1655 in this region, it was therefore surprising to detect a significant difference between symbiotic and nonsymbiotic strains in the ability to induce TLR5-mediated signaling. Flagellin of EcN, harboring the substantially longer HVR, induced significantly stronger TLR5-mediated signaling compared to the tested nonsymbiotic *E*. *coli* strains. This indicated an unexpected contribution of the HVR D2 and D3 domains to TLR5 activation.

Andersen-Niessen and colleagues reported that flagellin's N-terminal D1 domain predominantly determined its TLR5-stimulatory activity. However, this required additional contribution from the HVR D2/D3 and the CD1 domain [64]. This might indicate that HVR domains generally have a stronger impact on TLR5 activation than could have been gleaned from only the crystal structure of TLR5 and flagellin [30]. This structure shows zebrafish TLR5 in complex with *Salmonella* FliC, out of which 100 amino acids in the HVR of *Salmonella* are not structurally resolved. This indicates that parts of the HVR might be highly flexible and/or do not stably interact with TLR5, at least with zebrafish TLR5. The situation may be different for the murine TLR5 that was investigated here.

Since the D3 domain of *Salmonella* flagellin contributes to the stability of flagellin monomers [65], which is a prerequisite for TLR5 receptor activation, it might be possible that the HVR of symbiotic *E*. *coli* strains also positively regulates monomer stability, whereas mucus attachment, another function of flagellin, has been shown to be independent of the D3 region [46] and thus probably not relevant here. Although the precise molecular mechanism by which the HVR modulates TLR5 signaling thus remains to be established, our functional data warrant a more thorough exploration of this region, both functionally and structurally.

While extracellular flagellin is sensed by TLR5, leading to MyD88-dependent NFκB activation, flagellin present in the cytosol results in activation of the NLR Family CARD Domain Containing 4 (NLRC4) inflammasome [32]. NLRC4 activation leads to IL-1β and IL-18 secretion, and the NLRC4 inflammasome helps to discriminate harmful pathogens from beneficial commensals [66]. However, we assume that potential NLRC4-inflammasome–mediated effects play a neglectable role concerning the discrimination of symbiotic from nonsymbiotic *E*. *coli* strains because of two reasons: first, flagellin recognition by the NLRC4 inflammasome component NLR family, apoptosis inhibitory protein 5 (NAIP5) is crucially mediated by 35 amino acids within the flagellin CD0 domain [67], which is identical in all tested strains. Secondly, we demonstrated that flagellin-HVR–mediated symbiotic effects were completely abolished in TLR5-deficient mice and FEPs were sufficient to mediate symbiotic properties, therefore indicating that TLR5-dependent sensing of extracellular flagellin is far more important than a potential intracellular NLRC4 inflammasome activation in our system.

But how can it be explained that a stronger intestinal TLR5 signaling, induced by a symbiotic commensal, correlates with beneficial effects during DSS-induced colitis accompanied by lower systemic cytokine levels? Systemic loss of TLR5 signaling was shown to entail an overgrowth of flagellated members of the intestinal microbiota, promoting inflammatory conditions [48,56]. Furthermore, certain SNPs in the human *TLR5* gene are associated with higher incidence of IBDs [68,69]. Therefore, TLR5 expression and signaling seem to be necessary to maintain a balanced homeostatic microbiota composition and intestinal immune homeostasis. This effect has been mostly traced back to TLR5 signaling in IECs, which is suspected to lead to immune cell recruitment, which mediates clearance from bacteria breaching the mucus barrier [37]. However, using BMCM, we have identified intestinal LP CD11c^+^ cells as crucially contributing to the EcN-flagellin–mediated symbiotic effects. This observation is in line with findings that TLR5 on hematopoietic cells is involved in flagellin sensing [70] and flagellin-dependent activation of Th17 cells [56]. Furthermore, SNP-mediated differences in TLR5 signaling were observed to affect immune cells rather than epithelial cells.

Besides IECs, CD11c_+_ cells such as DCs are the most important TLR5-expressing cells at intestinal mucosal interfaces [71]. We demonstrated that symbiotic-flagellin–mediated beneficial effects on the progress of DSS-induced colitis was associated with increased serum levels of IL-22 as well as with a higher proportion of IL-22^+^ intDCs. In general, IL-22 contributes to restoration of intestinal homeostasis, promotes regeneration of damaged intestinal epithelium [72], modulates epithelial cell fucosylation [73], and induces the secretion of antimicrobial peptides [74,75] and mucins [76]. Therefore, it represents a pivotal cytokine in modulation of intestinal tissue responses during inflammatory processes. In intestinal tissue, IL-22 is mainly produced by ILC3 cells [59], CD4^+^ T cells [77], and, to a lesser extent, CD8^+^ T cells, T-cell receptor (TCR)γδ T cells, neutrophils, and natural killer (NK) cells [78,79]. ILC3 and CD4^+^ T cells secrete IL-22 mainly in response to IL-23 [58,59,77]. In this connection, intDCs play a decisive role in shaping the LP cytokine milieu in response to intestinal microbiota sensing. However, even intDCs themselves emerged as sources of gastrointestinal IL-22 [75,80,81] which is in line with our observations. Therefore, we hypothesize that symbiotic flagellin, bearing a longer HVR, leads to stronger TLR5 signaling in intDCs, resulting in enhanced IL-22 expression and thus contributing to maintenance of the intestinal barrier. Since disruption of the intestinal barrier provides a characteristic of DSS-induced colitis, IL-22 was demonstrated to play a pivotal role in counteracting inflammatory processes in this disease model [81–83]. However, and to date, we cannot completely exclude a merely indirect contribution of intDCs by modulating IL-22 secretion through ILC3 cells or T cells. This indirect influence might occur via secretion of ILC3- and T-cell–activating cytokines by CD11c^+^ cells other than intDCs. Nevertheless, our results indicate intDCs to be, at least partially, direct sources of IL-22 in response to different flagellins, which, in turn, might account for the observed distinct DSS-induced colitis phenotypes mediated by administration of different flagellins. Thus, paradoxically, a symbiotic-flagellin–mediated stronger TLR5 signaling based on its flagellin HVR structure might contribute to the observed lower inflammatory status of symbiotic-flagellin–administered DSS-treated mice.

Interestingly, flagellin-dependent TLR5 signaling was also demonstrated to be involved in the mediation of symbiotic properties of another gut commensal, *Roseburia hominis* [84], and may thus be a more common phenomenon than previously thought. However, the role of certain cell subtypes within the intestinal tissue mediating the decisive beneficial effects in response to flagellin still remains to be further elucidated. While we propose intDCs from the cLP to be the most important cell type to promote inflammation-preventing events during DSS-induced colitis, Uematsu and colleagues demonstrated CD11c^+^ cells from Peyer’s Patches (PPs) in the small intestine to be responsible for flagellin-induced IL-10 secretion, which might, in turn, contribute to homeostasis, while LP CD11c^+^ cells instead promote secretion of proinflammatory cytokines [85]. Therefore, both studies accentuate the contribution of intestinal CD11c^+^ cells on host immune homeostasis by sensing flagellin from luminal microbes, even though different CD11c^+^ cell populations emerged as crucial. Since our study focused on DSS-induced colitis, we did not further investigate cells from noncolonic gastrointestinal tissues. Uematsu and colleagues, on the other hand, did not investigate homeostasis-promoting mechanisms other than IL-10 secretion. Thus, both results are not necessarily contradictory. Nevertheless, more studies have to be conducted to uncover the role of CD11c+ cells all across the gastrointestinal tract in terms of flagellin-mediated immunological reactions.

In addition to local intestinal effects, the *E*. *coli*-flagellin-HVR–mediated impact on the progress of DSS-induced colitis in mice was accompanied by regulation of systemic expression of various pro- and anti-inflammatory cytokines. The most important pathology-promoting cytokines in IBD patients are IL-1β, IL-6, TNFα, IFNγ, and IL-17 [86]. We demonstrated that the EcN-administration–associated decrease of IL-1β, IL-17, IL-6, and TNFα serum levels in DSS-treated mice is abolished when an EcN deletion mutant is used that lacks large parts of its flagellin HVR. Therefore, the *E*. *coli* flagellin HVR seems to be involved in the regulation of systemic expression of such IBD-promoting cytokines. However, we think that this is instead a secondary effect, probably rooted in IL-22–mediated increased epithelial barrier integrity, thus preventing inflammation-driving translocation of luminal content.

Interestingly, Rakoff-Nahoum and colleagues demonstrated in 2004 that proper TLR2- and TLR4-signaling protects from DSS-induced mortality in microbiota-depleted mice, which is assumed to be necessary for ligand-dependent steady-state induction of protective factors [87]. Our data indicate that intestinal TLR5 signaling might also contribute to these fundamental homeostasis-preserving mechanisms.

Taken together, we propose the flagellin HVR structure may be a distinguishing marker for the classification of *E*. *coli* strains as either nonsymbiotic or symbiotic. Furthermore, we demonstrated that flagellin-mediated symbiotic effects were originated in the structure of the flagellin HVR, which, in turn, influences TLR5-mediated intracellular signaling in intestinal CD11c^+^ cells, accompanied by regulation of IBD-promoting cytokines. These insights might therefore offer new possibilities for drug development involving existing or custom-designed flagellin structures, especially for intestinal inflammatory disorders such as IBD.

## Materials and methods

### Ethics statement

In this study, we use WT C57BL/6 and TLR5-deficient animals as well as BMCM as described in the manuscript. This study was carried out in accordance with the principles of the Basel Declaration. Protocols and experiments involving mice were reviewed and approved by the responsible Institutional Review Committee and the local authorities (permit numbers: H3/18, H9/11, H5/10). All mice used in the experiments were killed by CO_2_ fumigation, as demanded by the responsible authorities. Mice were weighed daily, and a general checkup of the animals' constitution was performed daily. A weight loss of more than 20% compared to the start of the experiment is an indicator for unacceptable suffering, leading to immediate euthanization of the respective animal. Additionally, the general constitution of the mouse was evaluated according to a score sheet that was approved by the responsible authorities. Exceeding a certain score requires immediate euthanization of the mouse. However, no mouse used for the experiments in the submitted manuscript was required to be euthanized before the projected end of each experiment.

### Mice

C57BL/6N (WT) and *Tlr5*^−/−^ mice were bred and raised under SPF conditions in the animal facility at the University of Tübingen, Germany and did not provide any signs of spontaneous colitis. CD11cCre/R-DTA (ΔCD11c) [88] mice were bred under SPF conditions at the animal facility of the University of Erlangen. All animal experiments were reviewed and approved by the responsible authorities.

### Generation of BMCM

Mice were irradiated in a gamma cell (GammaCell 1000 Elite; Nordion International, Ottawa, ON, Canada) with 900 cGy (female) or 950 cGy (male). Six hours after irradiation, mice were injected intravenously with freshly isolated bone marrow cells (1 × 10^7^ cells/100 μL PBS) from donor mice. Bone marrow cells were isolated as described previously [41] with minor modifications. Mice were administered Cotrim E (26 mg/100 mL sterile drinking water, purchased from Ratiopharm, Ulm, Germany) for the first two weeks after irradiation. Six weeks after irradiation, blood was analyzed for successful reconstitution of the transplanted bone marrow by flow cytometry. BMCM were used for induction of DSS-induced colitis as described below.

### Bacteria

Mice were administered EcN, the isogenic mutant EcNΔ*fliC* [46], the nonsymbiotic MG1655 [89], and pathobiotic MPK [41,42,44]. Furthermore, an MG1655Δ*fliC* mutant was complemented with the *fliC* gene from EcN (MG1655*ΔfliC*::*fliC*(EcN)) and an EcNΔf*liC* mutant was complemented with the *fliC* gene from MPK (EcN*ΔfliC*::*fliC*(MPK)), and both were administered to mice. The EcNΔ*fliC*(HVR) mutant lacks large parts of the HVR within the *fliC* gene. All strains were grown at 37°C in LB broth under aerobic conditions.

### Generation of FEPs

Bacteria were grown in LB broth as described above. Bacteria were centrifuged at 4,000 rpm, and the cell pellet was resuspended in proteinase inhibitor cocktail (Roche, Basel, Switzerland) and shaken vigorously for 10 min. The suspension was centrifuged for 15 min (4,000 rpm, 4°C), and the supernatant was used as an FEP.

### Administration of live bacteria, FEPs, or recombinant flagellin to mice

We observed the progress of DSS-induced colitis in mice in response to treatment with viable bacteria, FEPs, or recombinant flagellin. Treatment with viable bacteria started 3 days prior to start of DSS administration by one-time intragastral gavage of a total of 10^8^ bacteria. Simultaneously, autoclaved drinking water was supplemented with viable bacteria at a final concentration of 10^8^ mL^−1^. 3 days after initial intragastral gavage, DSS was added to drinking water suspensions at a final concentration of 3.5% (w/v). Drinking water containing bacteria and DSS were renewed every 2 days until the end of the experiment. Colonization with viable bacteria was assessed by determination of CFUs, as described in the supplementary material. Administration of FEPs to DSS-treated mice was comparable: FEPs obtained from 10^7^ or 10^10^ bacteria were resuspended in 100 μL autoclaved drinking water and administered once by intragastral gavage 3 days prior to the start of DSS administration. Subsequent treatment with FEPs for the next 10 days was performed by generating drinking water suspensions containing FEPs from 10^7^ or 10^10^ in 100 mL drinking water. DSS was added to the drinking water suspension 3 days after initial FEP administration, and suspensions were renewed every 2 days. Recombinant flagellin was also started to be administered 3 d before start of DSS treatment. A total of 2 μg recombinant flagellin was administered daily by intragastral gavage for a total of 10 d. DSS treatment started 3 d after initial flagellin administration, and DSS solutions were renewed every 2 d.

### Generation of pure recombinant EcN flagellin preparations

Chemocompetent NiCo BL21 (D3) *E*. *coli* were transformed by heatshock with a pET19-b expression vector harboring the His-tagged sequence for EcN flagellin (FliC(EcN)). After recovery time, transformed bacteria were inoculated into ampicillin-containing LB medium and incubated at 37°C O/N. An aliquot of the O/N culture was grown until OD = 0.6. 200 μM. IPTG was added to induce lac-operon controlled protein expression of rfliC(EcN). Bacterial culture was grown O/N at 18°C and 90 rpm, and cells were harvested by centrifugation at 17,000 rpm and 4°C. The pellet was lysed for 15 min on ice using protease-inhibitor–and benzonase-containing lysis buffer (300 mM NaCl, 50 mM Tris-HCl [pH 8.0], 5 mM imidazole) at a ratio of 10 mL buffer per gram pellet followed by sonification on a Branson 250 sonifier (Branson Ultrasonics, Danbury, CT, USA). Lysates were centrifuged at 35,000 rpm for 45 min at 4°C. The 1 mL HisTrap Column (GE Healthcare Life Sciences, Marlborough, MA, USA) was equilibrated with equilibration buffer (300 mM NaCl, 50 mM Tris-HCl [pH 8.0]), and bacterial lysate was loaded on the column afterwards. Protein binding to the HisTrap Column was performed O/N. Next, columns were washed with various concentrations of elution buffer (300 mM NaCl, 50 mM Tris-HCl [pH 8.0], 500 mM imidazole), ranging from 3% to 100%. At 20% elution buffer, His-tagged rfliC(EcN) started to be eluted, and elute fractions were collected. Fractions were analyzed via SDS-PAGE and α-fliC western blotting as demonstrated in the supplementary material (S9 and S10 Figs).

### Isolation of LP cells and staining for flow cytometry

LP cells from the colon were isolated as published previously [41,57] with minor modifications. Cells were stained for viability using ViabilityStain (eBioscience, Thermo Fisher Scientific, Waltham, MA, USA) according to the manufacturer’s instruction. 2 × 10^6^ cells were incubated in DMEM (Gibco, Gaithersburg, MD, USA) supplemented with 10% FCS, 1% HEPES, 1% nonessential amino acids, 1% sodium pyruvate, 0.5% penicillin/streptomycin, 0.5% β-mercaptoethanol, and 2 μL leukocyte activation cocktail (BD Biosciences, San Jose, CA, USA) for 4 h at 37°C. Cells were washed and fixed with Cytofix/Cytoperm (BD Biosciences). Cells were washed in PBS/FCS containing 0.1% saponin and treated with Cytofix/Cytoperm (BD Biosciences) for 10 min at RT. Antibodies were diluted by factor 100 in PBS/FCS + 0.1% saponin and incubated with cells for 30 min at 4°C. Cells were washed twice, and flow cytometrical detection was performed subsequently.

### Antibodies, chemicals, and reagents

For flow cytometry, the following antibodies were used: α-mouse IL-22 (1H8PWSR; eBioscience), α-mouse IL-23p19 (N71-1183; BD Biosciences), α-mouse CD11c (HL3; BD Biosciences), α-mouse CD64 (X54-5/7.1; BD Biosciences), α-mouse CD45 (30-F11; BD Biosciences), α-mouse CD45R (RA3-6B2; BD Biosciences), and α-mouse Ly6G/C (GR-1/RB-68C5; BD Biosciences). Recombinant flagellin from MG1655 was obtained from MyBioSource (#MBS1265520; San Diego, CA, USA).

### DSS-induced colitis

Mice were administered live bacteria, recombinant flagellin, or FEP 3 days prior to challenging with 3.5% (w/v) DSS in drinking water and during the whole course of the experiment as described above. Body weight was determined on day 0 (start of DSS administration) as well as on days 3 to 7.

### Statistical analysis

For comparisons of two groups, a parametric Student *t* test was used for normally distributed values and nonparametric Mann–Whitney test elsewhere. For multiple comparison of more than two groups, one-way ANOVA was used for normally distributed values, and nonparametric Kruskal–Wallis test was used elsewhere. *p*-values are indicated in the figures. *p*-values < 0.05 were considered to be significant.

See Supplementary material S1 Text for additional information on experimental procedures.

## Supporting information

S1 Fig Determination of *E. coli* CFUs in the feces of DSS-treated mice. SPF C57BL/6 WT mice (a) and *Tlr5*^−/−^ mice (b) aged 6 to 8 weeks were administered 3.5% DSS in drinking water at day 0. Mice were additionally treated with EcN (DSS + EcN), MG1655 (DSS + MG1655), MPK (DSS + MPK), or the EcN Δ*fliC* deletion mutant (EcNΔ*fliC*) resuspended in DSS-containing drinking water at 10^8^ bacteria mL^−1^. At day 7 after start of DSS administration, feces were plated on Enterobacteriaceae-specific agar in serial dilutions and CFUs were determined by counting dark red colonies specific for *E*. *coli* strains. (a + b) The data underlying this figure can be found in S1 Data. CFU, colony-forming unit; DSS, dextran sodium sulphate; EcN, *E*. *coli* Nissle 1917; *fliC*, flagellin; MG1655, *E*. *coli* K12 MG1655; MPK, *E*. *coli* mpk; SPF, specific-pathogen–free; TLR, Toll-like receptor; WT, wild type.
(PNG)

S2 Fig EcN, MPK, and MG1655 express functional flagella. Right column: overnight cultures of MPK, EcN, and MG1655. Overnight bacterial culture was seeded in the middle of a swarming culture medium and incubated for 24 h. The inoculation spot is indicated by a red circle, and the borders of the swarming area are highlighted with a white scattered line. Left column: electron microscopy pictures (negative staining) of EcN (upper panel), MPK (middle panel), and MG1655 (lower panel) highlighting the respective flagellum (red arrows). MPK lost its flagella during the staining procedure and could be detected as the shed structure. The insert in the respective picture (left column, middle panel) shows an MPK bacterium. EcN, *E*. *coli* Nissle 1917; MG1655, *E*. *coli* K12 MG1655; MPK, *E*. *coli* mpk.
(PNG)

S3 Fig EcNΔ*fliC* does not express a functional flagellum. Right: overnight bacterial culture of EcNΔfliC was seeded in the middle of a swarming culture medium and incubated for 24 h. The inoculation spot is indicated by a red circle, and the borders of the swarming area are highlighted with a white scattered line. Left column: electron microscopy pictures (negative staining) of EcNΔfliC (highlighting the absence of flagella). EcN, *E*. *coli* Nissle 1917; *fliC*, flagellin.
(PNG)

S4 Fig Characterization of FEPs. EcN, MPK, and MG1655 were grown to OD100, and FEPs were generated as described in the main manuscript. (a) Silver staining of 10 μL FEP on an 8%–15% gradient SDS gel. (b) LAL test to determine endotoxin levels in FEPs. (c) Determination of overall protein concentration in FEPs using a bicinchoninic acid assisted assay. (d) Western blot of FliC of 10 μL FEPs using anti-flagellin antibody (ab93713; Abcam, Cambridge, UK). (e) Quantification of FliC concentrations in FEPs. Band intensities of FliC bands in western blots depicted in (d) were quantified. A standard curve of recombinant EcN FliC was generated and visualized with the same antibodies on the same blots. FliC concentrations were computed using the determined FliC band intensities in relation to a linear regression of the band intensities of FliC standard curve. (b + c + e) The data underlying this figure can be found in S1 Data. EcN, *E*. *coli* Nissle 1917; FEP, flagella-enriched preparation; *fliC*, flagellin; LAL, limulus amebocyte lysate; MG1655, *E*. *coli* K12 MG1655; MPK, *E*. *coli* mpk; OD, optical density.
(PNG)

S5 Fig Generation of *fliC* exchange mutant strains. Chromosomal exchange of *fliC* alleles was done by allelic exchange as described previously [90]. Upper panel: suicide plasmids were constructed by Gibson assembly according to standard protocols [91]. Lower panel: primers and plasmids for allelic exchange as well as resulting strains. *fliC*, flagellin.
(PNG)

S6 Fig MG1655ΔfliC::fliC(EcN) and EcNΔfliC::fliC(MPK) express a functional flagella. Right column: overnight bacterial culture of MG1655ΔfliC::fliC(EcN) and EcNΔfliC::fliC(MPK) exchange mutants were seeded in the middle of a swarming culture medium and incubated for 24 h. The inoculation spot is indicated by a red circle, and the borders of the swarming area are highlighted with a white scattered line. Left column: electron microscopy pictures of MG1655ΔfliC::fliC(EcN) and EcNΔfliC::fliC(MPK) highlighting the respective flagella (red arrow). EcN, *E*. *coli* Nissle 1917; *fliC*, flagellin; MG1655, *E*. *coli* K12 MG1655; MPK, *E*. *coli* mpk.
(PNG)

S7 Fig EcNΔflic(HVR) expresses a shorter flagella compared to WT EcN. Left panel: EM pictures of EcNΔfliC(HVR) deletion mutants highlighting the flagella (red arrow). Right panel: EM-assisted determination of flagella lengths. Each white dot represents one detected flagellum in EM pictures. The data underlying this figure can be found in S1 Data. EcN, *E*. *coli* Nissle 1917; EM, electron microscopy; *fliC*, flagellin; HVR, hypervariable region; WT, wild type.
(PNG)

S8 Fig Schematic illustration of generation of BMCM. (A) *Tlr5*^−/−^ → WT mice by irradiation of C57BL/6×WT-CD45.1–expressing mice transplanted with bone marrow of C57BL/6×*Tlr5*^−/−^-CD45.2–expressing mice and (B) WT → *Tlr5*^−/−^ mice by irradiation of C57BL/6×*Tlr5*^−/−^-CD45.2–expressing mice transplanted with bone marrow of C57BL/6×WT-CD45.1–expressing mice. (C) Irradiated C57BL/6-CD45.2 mice transplanted with C57BL/6-CD45.1 bone marrow (WT → WT), (D) *Tlr5*^−/−^CD45.2 mice transplanted with *Tlr5*^−/−^-CD45.2 bone marrow (*Tlr5*^−/−^ → *Tlr5*^−/−^). Successful transplantation was monitored by flow cytometry analysis of blood samples stained with antibodies against CD45.1 and CD45.2. Figures show means ± SD of 4 to 9 mice per experiment. BMCM, bone-marrow–chimeric mice; TLR, Toll-like receptor; WT, wild type.
(PNG)

S9 Fig Validation of rFliC(EcN) purity. Coomassi-stained 4%–15% gradient gel of all collected eluted fractions after elution from HisTrap columns using elution buffer (300 mM NaCl, 50 mM Tris-HCl [pH 8.0], 500 mM imidazole) at concentrations from 20% to 100%. EcN, *E*. *coli* Nissle 1917; *fliC*, flagellin; rfliC(EcN), recombinant flagellin from EcN.
(PNG)

S10 Fig Western blot of rFliC(EcN). Western blots against the His-tags of rFliC(EcN) (left panel) and FliC (right panel) were performed to verify the proper expression of the recombinant protein. Cell lysates before IPTG-assisted induction of protein expression (preinduction), after IPTG-assisted induction (postinduction), the collected elutes from the HisTrap column (Elute) (see S9 Fig), and a previously purified MS-controlled rFliC(EcN) as positive control were loaded on a 4%–15% gradient gel, and western blots were performed as described. EcN, *E*. *coli* Nissle 1917; *fliC*, flagellin; MS, mass spectrometry; rfliC(EcN), recombinant flagellin from EcN.
(PNG)

S1 Table Detailed statistical analysis of the values depicted in Fig 1E as determined by one-way ANOVA. (DOCX)

S2 Table Statistical analysis of cytokine serum levels in DSS-treated mice. *p*-values were computed using nonparametric Kruskal–Wallis test. DSS, dextran sodium sulphate.
(DOCX)

S1 Data Raw data underlying the following figures: Fig 1, Fig 2, Fig 4, Fig 5, Fig 6, S1 Fig, S4 Fig, S7 Fig. (XLSX)

S1 Text Supplementary methods. (DOCX)

## Abbreviations

BMCM: bone-marrow–chimeric mice
CD: C-terminal domain
CFU: colony-forming unit
cLP: colonic LP
DC: dendritic cell
DSS: dextran sodium sulphate
EcN: *E*. *coli* Nissle 1917
FEP: flagella-enriched preparation
fliC: flagellin
FSC: forward scatter
GM-CSF: granulocyte-macrophage colony-stimulating factor
HCS: histological colitis score
HE: hematoxylin–eosin
HVR: hypervariable region
IBD: Inflammatory Bowel Disease
IEC: intestinal epithelial cell
IFN: interferon
IL: interleukin
ILC: innate lymphoid cell
intDC: intestinal DC
LP: lamina propria
MAFFT: Multiple Alignment using Fast Fourier Transform
MAMP: microbe-associated molecular pattern
MCP: monocyte chemoattractant protein
MG1655: *E*. *coli* K12 MG1655
MOI: multiplicity of infection
MPK: *E*. *coli* mpk
mTLR5-HEK293 cell: mouse-TLR5–expressing human embryonic kidney 293 cell
MyD: myeloid differentiation primary response
NAIP: NLR family, apoptosis inhibitory protein 5
ND: N-terminal domain
NFκB: nuclear factor “kappa-light-chain–enhancer” of activated B-cells
NK: natural killer
NLRC: NLR Family CARD Domain Containing
PP: Peyer’s Patch
PRR: pattern-recognition receptor
rfliC(EcN): recombinant flagellin from EcN
rfliC(K12): recombinant flagellin from MG1655
SPF: specific-pathogen–free
TCRγδ: T-cell receptor γδ
Th: T helper
TLR: Toll-like receptor
TNF: tumor necrosis factor
UC: ulcerative colitis
WT: wild type

## References

[1] Kamada N, Seo SU, Chen GY, Nunez G. Role of the gut microbiota in immunity and inflammatory disease. Nat Rev Immunol. 2013;13: 321–335. 10.1038/nri3430 23618829

[2] Wassenaar TM. Insights from 100 Years of Research with Probiotic E. Coli. Eur J Microbiol Immunol (Bp). 2016;6: 147–161. 27766164 10.1556/1886.2016.00029 PMC5063008

[3] Hill MJ, Drasar BS. The normal colonic bacterial flora. Gut. 1975;16: 318–323. 10.1136/gut.16.4.318 1093952 PMC1410930

[4] Khan AA, Khan Z, Malik A, Kalam MA, Cash P, et al Colorectal cancer-inflammatory bowel disease nexus and felony of Escherichia coli. Life Sci. 2017;180: 60–67. 10.1016/j.lfs.2017.05.016 28506682

[5] Baumgart M, Dogan B, Rishniw M, Weitzman G, Bosworth B, et al Culture independent analysis of ileal mucosa reveals a selective increase in invasive Escherichia coli of novel phylogeny relative to depletion of Clostridiales in Crohn's disease involving the ileum. ISME J. 2007;1: 403–418. 10.1038/ismej.2007.52 18043660

[6] Frank DN, St Amand AL, Feldman RA, Boedeker EC, Harpaz N, et al Molecular-phylogenetic characterization of microbial community imbalances in human inflammatory bowel diseases. Proc Natl Acad Sci U S A. 2007;104: 13780–13785. 10.1073/pnas.0706625104 17699621 PMC1959459

[7] Lepage P, Hasler R, Spehlmann ME, Rehman A, Zvirbliene A, et al Twin study indicates loss of interaction between microbiota and mucosa of patients with ulcerative colitis. Gastroenterology. 2011;141: 227–236. 10.1053/j.gastro.2011.04.011 21621540

[8] Scaldaferri F, Gerardi V, Mangiola F, Lopetuso LR, Pizzoferrato M, et al Role and mechanisms of action of Escherichia coli Nissle 1917 in the maintenance of remission in ulcerative colitis patients: An update. World J Gastroenterol. 2016;22: 5505–5511. 10.3748/wjg.v22.i24.5505 27350728 PMC4917610

[9] Kruis W, Schutz E, Fric P, Fixa B, Judmaier G, et al Double-blind comparison of an oral Escherichia coli preparation and mesalazine in maintaining remission of ulcerative colitis. Aliment Pharmacol Ther. 1997;11: 853–858. 9354192 10.1046/j.1365-2036.1997.00225.x

[10] Rembacken BJ, Snelling AM, Hawkey PM, Chalmers DM, Axon AT. Non-pathogenic Escherichia coli versus mesalazine for the treatment of ulcerative colitis: a randomised trial. Lancet. 1999;354: 635–639. 10.1016/s0140-6736(98)06343-0 10466665

[11] Henker J, Muller S, Laass MW, Schreiner A, Schulze J. Probiotic Escherichia coli Nissle 1917 (EcN) for successful remission maintenance of ulcerative colitis in children and adolescents: an open-label pilot study. Z Gastroenterol. 2008;46: 874–875. 10.1055/s-2008-1027463 18810672

[12] Lasaro MA, Salinger N, Zhang J, Wang Y, Zhong Z, et al F1C fimbriae play an important role in biofilm formation and intestinal colonization by the Escherichia coli commensal strain Nissle 1917. Appl Environ Microbiol. 2009;75: 246–251. 10.1128/AEM.01144-08 18997018 PMC2612203

[13] Wehkamp J, Harder J, Wehkamp K, Wehkamp-von Meissner B, Schlee M, et al NF-kappaB- and AP-1-mediated induction of human beta defensin-2 in intestinal epithelial cells by Escherichia coli Nissle 1917: a novel effect of a probiotic bacterium. Infect Immun. 2004;72: 5750–5758. 10.1128/IAI.72.10.5750-5758.2004 15385474 PMC517557

[14] Zyrek AA, Cichon C, Helms S, Enders C, Sonnenborn U, et al Molecular mechanisms underlying the probiotic effects of Escherichia coli Nissle 1917 involve ZO-2 and PKCzeta redistribution resulting in tight junction and epithelial barrier repair. Cell Microbiol. 2007;9: 804–816. 10.1111/j.1462-5822.2006.00836.x 17087734

[15] Sassone-Corsi M, Nuccio SP, Liu H, Hernandez D, Vu CT, et al Microcins mediate competition among Enterobacteriaceae in the inflamed gut. Nature. 2016;540: 280–283. 10.1038/nature20557 27798599 PMC5145735

[16] Sturm A, Rilling K, Baumgart DC, Gargas K, Abou-Ghazale T, et al Escherichia coli Nissle 1917 distinctively modulates T-cell cycling and expansion via toll-like receptor 2 signaling. Infect Immun. 2005;73: 1452–1465. 10.1128/IAI.73.3.1452-1465.2005 15731043 PMC1064918

[17] Jiminez JA, Uwiera TC, Douglas Inglis G, Uwiera RR. Animal models to study acute and chronic intestinal inflammation in mammals. Gut Pathog. 2015;7: 29 10.1186/s13099-015-0076-y 26561503 PMC4641401

[18] Fang K, Bruce M, Pattillo CB, Zhang S, Stone R 2nd, et al Temporal genomewide expression profiling of DSS colitis reveals novel inflammatory and angiogenesis genes similar to ulcerative colitis. Physiol Genomics. 2011;43: 43–56. 10.1152/physiolgenomics.00138.2010 20923862 PMC3026350

[19] Kanwar B, Gao DW, Hwang AB, Grenert JP, Williams SP, et al In vivo imaging of mucosal CD4+ T cells using single photon emission computed tomography in a murine model of colitis. J Immunol Methods. 2008;329: 21–30. 10.1016/j.jim.2007.09.008 17964595 PMC2683264

[20] Dieleman LA, Palmen MJ, Akol H, Bloemena E, Pena AS, et al Chronic experimental colitis induced by dextran sulphate sodium (DSS) is characterized by Th1 and Th2 cytokines. Clin Exp Immunol. 1998;114: 385–391. 10.1046/j.1365-2249.1998.00728.x 9844047 PMC1905133

[21] Kim TW, Seo JN, Suh YH, Park HJ, Kim JH, et al Involvement of lymphocytes in dextran sulfate sodium-induced experimental colitis. World J Gastroenterol. 2006;12: 302–305. 10.3748/wjg.v12.i2.302 16482634 PMC4066043

[22] Teahon K, Smethurst P, Pearson M, Levi AJ, Bjarnason I. The effect of elemental diet on intestinal permeability and inflammation in Crohn's disease. Gastroenterology. 1991;101: 84–89. 1904381 10.1016/0016-5085(91)90463-u

[23] Keshavarzian A, Price YE, Peters AM, Lavender JP, Wright NA, et al Specificity of indium-111 granulocyte scanning and fecal excretion measurement in inflammatory bowel disease—an autoradiographic study. Dig Dis Sci. 1985;30: 1156–1160. 4064866 10.1007/BF01314050

[24] Costa F, Mumolo MG, Ceccarelli L, Bellini M, Romano MR, et al Calprotectin is a stronger predictive marker of relapse in ulcerative colitis than in Crohn's disease. Gut. 2005;54: 364–368. 10.1136/gut.2004.043406 15710984 PMC1774401

[25] Melgar S, Karlsson L, Rehnstrom E, Karlsson A, Utkovic H, et al Validation of murine dextran sulfate sodium-induced colitis using four therapeutic agents for human inflammatory bowel disease. Int Immunopharmacol. 2008;8: 836–844. 10.1016/j.intimp.2008.01.036 18442787

[26] Claes IJ, De Keersmaecker SC, Vanderleyden J, Lebeer S. Lessons from probiotic-host interaction studies in murine models of experimental colitis. Mol Nutr Food Res. 2011;55: 1441–1453. 10.1002/mnfr.201100139 21796777

[27] Kawasaki T, Kawai T. Toll-like receptor signaling pathways. Front Immunol. 2014;5: 461 10.3389/fimmu.2014.00461 25309543 PMC4174766

[28] Song WS, Jeon YJ, Namgung B, Hong M, Yoon SI. A conserved TLR5 binding and activation hot spot on flagellin. Sci Rep. 2017;7: 40878 10.1038/srep40878 28106112 PMC5247705

[29] Smith KD, Andersen-Nissen E, Hayashi F, Strobe K, Bergman MA, et al Toll-like receptor 5 recognizes a conserved site on flagellin required for protofilament formation and bacterial motility. Nat Immunol. 2003;4: 1247–1253. 10.1038/ni1011 14625549

[30] Yoon SI, Kurnasov O, Natarajan V, Hong M, Gudkov AV, et al Structural basis of TLR5-flagellin recognition and signaling. Science. 2012;335: 859–864. 10.1126/science.1215584 22344444 PMC3406927

[31] Ramos HC, Rumbo M, Sirard JC. Bacterial flagellins: mediators of pathogenicity and host immune responses in mucosa. Trends Microbiol. 2004;12: 509–517. 10.1016/j.tim.2004.09.002 15488392

[32] Zhao Y, Yang J, Shi J, Gong YN, Lu Q, et al The NLRC4 inflammasome receptors for bacterial flagellin and type III secretion apparatus. Nature. 2011;477: 596–600. 10.1038/nature10510 21918512

[33] Lodes MJ, Cong Y, Elson CO, Mohamath R, Landers CJ, et al Bacterial flagellin is a dominant antigen in Crohn disease. J Clin Invest. 2004;113: 1296–1306. 10.1172/JCI20295 15124021 PMC398429

[34] Meena NK, Ahuja V, Meena K, Paul J. Association of TLR5 gene polymorphisms in ulcerative colitis patients of north India and their role in cytokine homeostasis. PLoS ONE. 2015;10: e0120697 10.1371/journal.pone.0120697 25789623 PMC4366177

[35] Klimosch SN, Forsti A, Eckert J, Knezevic J, Bevier M, et al Functional TLR5 genetic variants affect human colorectal cancer survival. Cancer Res. 2013;73: 7232–7242. 10.1158/0008-5472.CAN-13-1746 24154872

[36] Singh V, Yeoh BS, Carvalho F, Gewirtz AT, Vijay-Kumar M. Proneness of TLR5 deficient mice to develop colitis is microbiota dependent. Gut Microbes. 2015;6: 279–283. 10.1080/19490976.2015.1060390 26067589 PMC4615783

[37] Chassaing B, Ley RE, Gewirtz AT. Intestinal epithelial cell toll-like receptor 5 regulates the intestinal microbiota to prevent low-grade inflammation and metabolic syndrome in mice. Gastroenterology. 2014;147: 1363–1377 e1317. 10.1053/j.gastro.2014.08.033 25172014 PMC4253564

[38] Grabig A, Paclik D, Guzy C, Dankof A, Baumgart DC, et al Escherichia coli strain Nissle 1917 ameliorates experimental colitis via toll-like receptor 2- and toll-like receptor 4-dependent pathways. Infect Immun. 2006;74: 4075–4082. 10.1128/IAI.01449-05 16790781 PMC1489743

[39] Garrido-Mesa N, Utrilla P, Comalada M, Zorrilla P, Garrido-Mesa J, et al The association of minocycline and the probiotic Escherichia coli Nissle 1917 results in an additive beneficial effect in a DSS model of reactivated colitis in mice. Biochem Pharmacol. 2011;82: 1891–1900. 10.1016/j.bcp.2011.09.004 21930116

[40] Olier M, Marcq I, Salvador-Cartier C, Secher T, Dobrindt U, et al Genotoxicity of Escherichia coli Nissle 1917 strain cannot be dissociated from its probiotic activity. Gut Microbes. 2012;3: 501–509. 10.4161/gmic.21737 22895085 PMC3495787

[41] Steimle A, Gronbach K, Beifuss B, Schafer A, Harmening R, et al Symbiotic gut commensal bacteria act as host cathepsin S activity regulators. J Autoimmun. 2016;75: 82–95. 10.1016/j.jaut.2016.07.009 27484364

[42] Waidmann M, Bechtold O, Frick JS, Lehr HA, Schubert S, et al *Bacteroides vulgatus* protects against *Escherichia coli*-induced colitis in gnotobiotic interleukin-2-deficient mice. Gastroenterology. 2003;125: 162–177. 12851881 10.1016/s0016-5085(03)00672-3

[43] Muller M, Fink K, Geisel J, Kahl F, Jilge B, et al Intestinal colonization of IL-2 deficient mice with non-colitogenic B. vulgatus prevents DC maturation and T-cell polarization. PLoS ONE. 2008;3: e2376 10.1371/journal.pone.0002376 18545662 PMC2398772

[44] Frick JS, Zahir N, Muller M, Kahl F, Bechtold O, et al Colitogenic and non-colitogenic commensal bacteria differentially trigger DC maturation and Th cell polarization: an important role for IL-6. Eur J Immunol. 2006;36: 1537–1547. 10.1002/eji.200635840 16708404

[45] Secher T, Kassem S, Benamar M, Bernard I, Boury M, et al Oral Administration of the Probiotic Strain Escherichia coli Nissle 1917 Reduces Susceptibility to Neuroinflammation and Repairs Experimental Autoimmune Encephalomyelitis-Induced Intestinal Barrier Dysfunction. Front Immunol. 2017;8: 1096 10.3389/fimmu.2017.01096 28959254 PMC5603654

[46] Troge A, Scheppach W, Schroeder BO, Rund SA, Heuner K, et al More than a marine propeller—the flagellum of the probiotic Escherichia coli strain Nissle 1917 is the major adhesin mediating binding to human mucus. Int J Med Microbiol. 2012;302: 304–314. 10.1016/j.ijmm.2012.09.004 23131416

[47] Vijay-Kumar M, Aitken JD, Carvalho FA, Cullender TC, Mwangi S, et al Metabolic syndrome and altered gut microbiota in mice lacking Toll-like receptor 5. Science. 2010;328: 228–231. 10.1126/science.1179721 20203013 PMC4714868

[48] Vijay-Kumar M, Sanders CJ, Taylor RT, Kumar A, Aitken JD, et al Deletion of TLR5 results in spontaneous colitis in mice. J Clin Invest. 2007;117: 3909–3921. 10.1172/JCI33084 18008007 PMC2075480

[49] Ubeda C, Lipuma L, Gobourne A, Viale A, Leiner I, et al Familial transmission rather than defective innate immunity shapes the distinct intestinal microbiota of TLR-deficient mice. J Exp Med. 2012;209: 1445–1456. 10.1084/jem.20120504 22826298 PMC3409501

[50] Yonekura K, Maki-Yonekura S, Namba K. Complete atomic model of the bacterial flagellar filament by electron cryomicroscopy. Nature. 2003;424: 643–650. 10.1038/nature01830 12904785

[51] Yonekura K, Maki-Yonekura S, Namba K. Structure analysis of the flagellar cap-filament complex by electron cryomicroscopy and single-particle image analysis. J Struct Biol. 2001;133: 246–253. 10.1006/jsbi.2000.4345 11472095

[52] Jeengar MK, Thummuri D, Magnusson M, Naidu VGM, Uppugunduri S. Uridine Ameliorates Dextran Sulfate Sodium (DSS)-Induced Colitis in Mice. Sci Rep. 2017;7: 3924 10.1038/s41598-017-04041-9 28634361 PMC5478663

[53] Alex P, Zachos NC, Nguyen T, Gonzales L, Chen TE, et al Distinct cytokine patterns identified from multiplex profiles of murine DSS and TNBS-induced colitis. Inflamm Bowel Dis. 2009;15: 341–352. 10.1002/ibd.20753 18942757 PMC2643312

[54] Marques R, Boneca IG. Expression and functional importance of innate immune receptors by intestinal epithelial cells. Cell Mol Life Sci. 2011;68: 3661–3673. 10.1007/s00018-011-0829-9 21984599 PMC11115018

[55] Price AE, Shamardani K, Lugo KA, Deguine J, Roberts AW, et al A Map of Toll-like Receptor Expression in the Intestinal Epithelium Reveals Distinct Spatial, Cell Type-Specific, and Temporal Patterns. Immunity. 2018;49: 560–575 e566. 10.1016/j.immuni.2018.07.016 30170812 PMC6152941

[56] Liu H, Chen F, Wu W, Cao AT, Xue X, et al TLR5 mediates CD172alpha(+) intestinal lamina propria dendritic cell induction of Th17 cells. Sci Rep. 2016;6: 22040 10.1038/srep22040 26907705 PMC4764953

[57] Gronbach K, Flade I, Holst O, Lindner B, Ruscheweyh HJ, et al Endotoxicity of lipopolysaccharide as a determinant of T-cell-mediated colitis induction in mice. Gastroenterology. 2014;146: 765–775. 10.1053/j.gastro.2013.11.033 24269927

[58] Takatori H, Kanno Y, Watford WT, Tato CM, Weiss G, et al Lymphoid tissue inducer-like cells are an innate source of IL-17 and IL-22. J Exp Med. 2009;206: 35–41. 10.1084/jem.20072713 19114665 PMC2626689

[59] Cella M, Fuchs A, Vermi W, Facchetti F, Otero K, et al A human natural killer cell subset provides an innate source of IL-22 for mucosal immunity. Nature. 2009;457: 722–725. 10.1038/nature07537 18978771 PMC3772687

[60] Scholtens PA, Oozeer R, Martin R, Amor KB, Knol J. The early settlers: intestinal microbiology in early life. Annu Rev Food Sci Technol. 2012;3: 425–447. 10.1146/annurev-food-022811-101120 22224552

[61] Pagnini C, Saeed R, Bamias G, Arseneau KO, Pizarro TT, et al Probiotics promote gut health through stimulation of epithelial innate immunity. Proc Natl Acad Sci U S A. 2010;107: 454–459. 10.1073/pnas.0910307107 20018654 PMC2806692

[62] Fabrega MJ, Rodriguez-Nogales A, Garrido-Mesa J, Algieri F, Badia J, et al Intestinal Anti-inflammatory Effects of Outer Membrane Vesicles from Escherichia coli Nissle 1917 in DSS-Experimental Colitis in Mice. Front Microbiol. 2017;8: 1274 10.3389/fmicb.2017.01274 28744268 PMC5504144

[63] Cheng K, Sloan A, Peterson L, McCorrister S, Robinson A, et al Comparative study of traditional flagellum serotyping and liquid chromatography-tandem mass spectrometry-based flagellum typing with clinical Escherichia coli isolates. J Clin Microbiol. 2014;52: 2275–2278. 10.1128/JCM.00174-14 24671787 PMC4042737

[64] Andersen-Nissen E, Smith KD, Strobe KL, Barrett SL, Cookson BT, et al Evasion of Toll-like receptor 5 by flagellated bacteria. Proc Natl Acad Sci U S A. 2005;102: 9247–9252. 10.1073/pnas.0502040102 15956202 PMC1166605

[65] Muskotal A, Seregelyes C, Sebestyen A, Vonderviszt F. Structural basis for stabilization of the hypervariable D3 domain of Salmonella flagellin upon filament formation. J Mol Biol. 2010;403: 607–615. 10.1016/j.jmb.2010.09.024 20868693

[66] Franchi L, Kamada N, Nakamura Y, Burberry A, Kuffa P, et al NLRC4-driven production of IL-1beta discriminates between pathogenic and commensal bacteria and promotes host intestinal defense. Nat Immunol. 2012;13: 449–456. 10.1038/ni.2263 22484733 PMC3361590

[67] Lightfield KL, Persson J, Brubaker SW, Witte CE, von Moltke J, et al Critical function for Naip5 in inflammasome activation by a conserved carboxy-terminal domain of flagellin. Nat Immunol. 2008;9: 1171–1178. 10.1038/ni.1646 18724372 PMC2614210

[68] Gewirtz AT, Vijay-Kumar M, Brant SR, Duerr RH, Nicolae DL, et al Dominant-negative TLR5 polymorphism reduces adaptive immune response to flagellin and negatively associates with Crohn's disease. Am J Physiol Gastrointest Liver Physiol. 2006;290: G1157–1163. 10.1152/ajpgi.00544.2005 16439468

[69] Leifer CA, McConkey C, Li S, Chassaing B, Gewirtz AT, et al Linking genetic variation in human Toll-like receptor 5 genes to the gut microbiome's potential to cause inflammation. Immunol Lett. 2014;162: 3–9. 10.1016/j.imlet.2014.07.017 25284610 PMC4259828

[70] Sanders CJ, Moore DA 3rd, Williams IR, Gewirtz AT. Both radioresistant and hemopoietic cells promote innate and adaptive immune responses to flagellin. J Immunol. 2008;180: 7184–7192. 10.4049/jimmunol.180.11.7184 18490717

[71] Hayashi F, Smith KD, Ozinsky A, Hawn TR, Yi EC, et al The innate immune response to bacterial flagellin is mediated by Toll-like receptor 5. Nature. 2001;410: 1099–1103. 10.1038/35074106 11323673

[72] Dudakov JA, Hanash AM, van den Brink MR. Interleukin-22: immunobiology and pathology. Annu Rev Immunol. 2015;33: 747–785. 10.1146/annurev-immunol-032414-112123 25706098 PMC4407497

[73] Goto Y, Obata T, Kunisawa J, Sato S, Ivanov II, et al Innate lymphoid cells regulate intestinal epithelial cell glycosylation. Science. 2014;345: 1254009 10.1126/science.1254009 25214634 PMC4774895

[74] Wolk K, Kunz S, Witte E, Friedrich M, Asadullah K, et al IL-22 increases the innate immunity of tissues. Immunity. 2004;21: 241–254. 10.1016/j.immuni.2004.07.007 15308104

[75] Zheng Y, Valdez PA, Danilenko DM, Hu Y, Sa SM, et al Interleukin-22 mediates early host defense against attaching and effacing bacterial pathogens. Nat Med. 2008;14: 282–289. 10.1038/nm1720 18264109

[76] Sugimoto K, Ogawa A, Mizoguchi E, Shimomura Y, Andoh A, et al IL-22 ameliorates intestinal inflammation in a mouse model of ulcerative colitis. J Clin Invest. 2008;118: 534–544. 10.1172/JCI33194 18172556 PMC2157567

[77] Liang SC, Tan XY, Luxenberg DP, Karim R, Dunussi-Joannopoulos K, et al Interleukin (IL)-22 and IL-17 are coexpressed by Th17 cells and cooperatively enhance expression of antimicrobial peptides. J Exp Med. 2006;203: 2271–2279. 10.1084/jem.20061308 16982811 PMC2118116

[78] Zhou G, Yu L, Fang L, Yang W, Yu T, et al CD177(+) neutrophils as functionally activated neutrophils negatively regulate IBD. Gut. 2018;67: 1052–1063. 10.1136/gutjnl-2016-313535 28468761

[79] Sonnenberg GF, Fouser LA, Artis D. Border patrol: regulation of immunity, inflammation and tissue homeostasis at barrier surfaces by IL-22. Nat Immunol. 2011;12: 383–390. 10.1038/ni.2025 21502992

[80] Mann ER, Bernardo D, Ng SC, Rigby RJ, Al-Hassi HO, et al Human gut dendritic cells drive aberrant gut-specific t-cell responses in ulcerative colitis, characterized by increased IL-4 production and loss of IL-22 and IFNgamma. Inflamm Bowel Dis. 2014;20: 2299–2307. 10.1097/MIB.0000000000000223 25397892

[81] Pickert G, Neufert C, Leppkes M, Zheng Y, Wittkopf N, et al STAT3 links IL-22 signaling in intestinal epithelial cells to mucosal wound healing. J Exp Med. 2009;206: 1465–1472. 10.1084/jem.20082683 19564350 PMC2715097

[82] Zenewicz LA, Yancopoulos GD, Valenzuela DM, Murphy AJ, Stevens S, et al Innate and adaptive interleukin-22 protects mice from inflammatory bowel disease. Immunity. 2008;29: 947–957. 10.1016/j.immuni.2008.11.003 19100701 PMC3269819

[83] Macho-Fernandez E, Koroleva EP, Spencer CM, Tighe M, Torrado E, et al Lymphotoxin beta receptor signaling limits mucosal damage through driving IL-23 production by epithelial cells. Mucosal Immunol. 2015;8: 403–413. 10.1038/mi.2014.78 25183367 PMC4364000

[84] Patterson AM, Mulder IE, Travis AJ, Lan A, Cerf-Bensussan N, et al Human Gut Symbiont Roseburia hominis Promotes and Regulates Innate Immunity. Front Immunol. 2017;8: 1166 10.3389/fimmu.2017.01166 29018440 PMC5622956

[85] Uematsu S, Jang MH, Chevrier N, Guo Z, Kumagai Y, et al Detection of pathogenic intestinal bacteria by Toll-like receptor 5 on intestinal CD11c+ lamina propria cells. Nat Immunol. 2006;7: 868–874. 10.1038/ni1362 16829963

[86] Strober W, Fuss IJ. Proinflammatory cytokines in the pathogenesis of inflammatory bowel diseases. Gastroenterology. 2011;140: 1756–1767. 10.1053/j.gastro.2011.02.016 21530742 PMC3773507

[87] Rakoff-Nahoum S, Paglino J, Eslami-Varzaneh F, Edberg S, Medzhitov R. Recognition of commensal microflora by toll-like receptors is required for intestinal homeostasis. Cell. 2004;118: 229–241. 10.1016/j.cell.2004.07.002 15260992

[88] Ohnmacht C, Pullner A, King SB, Drexler I, Meier S, et al Constitutive ablation of dendritic cells breaks self-tolerance of CD4 T cells and results in spontaneous fatal autoimmunity. J Exp Med. 2009;206: 549–559. 10.1084/jem.20082394 19237601 PMC2699126

[89] Smith SN, Hagan EC, Lane MC, Mobley HL. Dissemination and systemic colonization of uropathogenic Escherichia coli in a murine model of bacteremia. MBio. 2010;1(5): e00262–10. 10.1128/mBio.00262-10 21116344 PMC2993011

[90] Kaniga K, Bossio JC, Galan JE. The Salmonella typhimurium invasion genes invF and invG encode homologues of the AraC and PulD family of proteins. Mol Microbiol. 1994;13:555–68. 7997169 10.1111/j.1365-2958.1994.tb00450.x

[91] Gibson DG, Young L, Chuang RY, et al Enzymatic assembly of DNA molecules up to several hundred kilobases. Nat Methods. 2009;6:343–5. 10.1038/nmeth.1318 19363495

