# Supplementary figures and images for: Flagellin hypervariable region determines symbiotic properties of commensal Escherichia coli strains
Source: PLoS Biol. 2019 Jun 17;17(6):e3000334. doi: 10.1371/journal.pbio.3000334 (PMC6597123; doi:10.1371/journal.pbio.3000334)

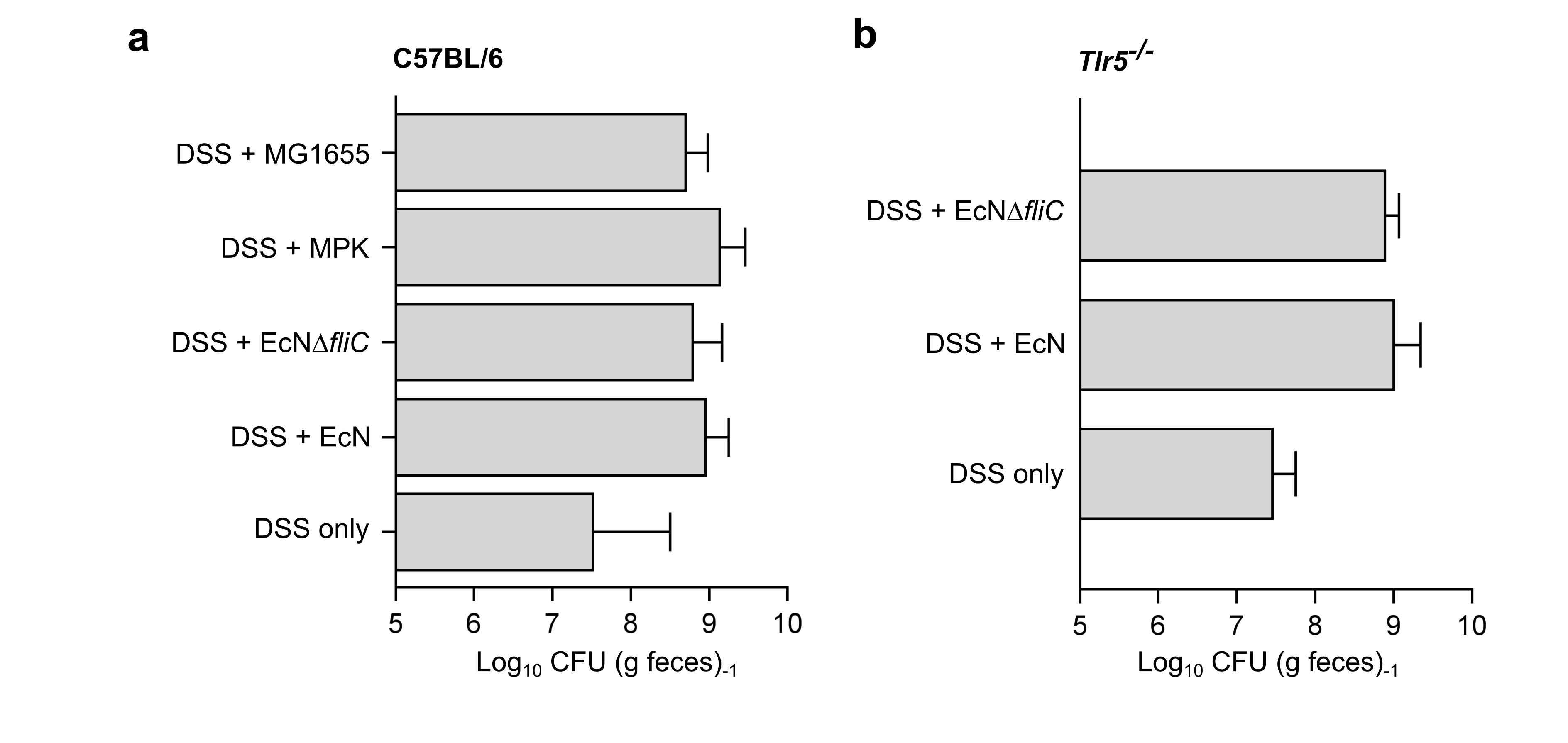

Supplement: S1 Fig — SPF C57BL/6 WT mice (a) and Tlr5−/− mice (b) aged 6 to 8 weeks were administered 3.5% DSS in drinking water at day 0. Mice were additionally treated with EcN (DSS + EcN), MG1655 (DSS + MG1655), MPK (DSS + MPK), or the EcN ΔfliC deletion mutant (EcNΔfliC) resuspended in DSS-containing drinking water at 108 bacteria mL−1. At day 7 after start of DSS administration, feces were plated on Enterobacteriaceae-specific agar in serial dilutions and CFUs were determined by counting dark red colonies specific for E. coli strains. (a + b) The data underlying this figure can be found in S1 Data. CFU, colony-forming unit; DSS, dextran sodium sulphate; EcN, E. coli Nissle 1917; fliC, flagellin; MG1655, E. coli K12 MG1655; MPK, E. coli mpk; SPF, specific-pathogen–free; TLR, Toll-like receptor; WT, wild type. (PNG) [file pbio.3000334.s001.png]

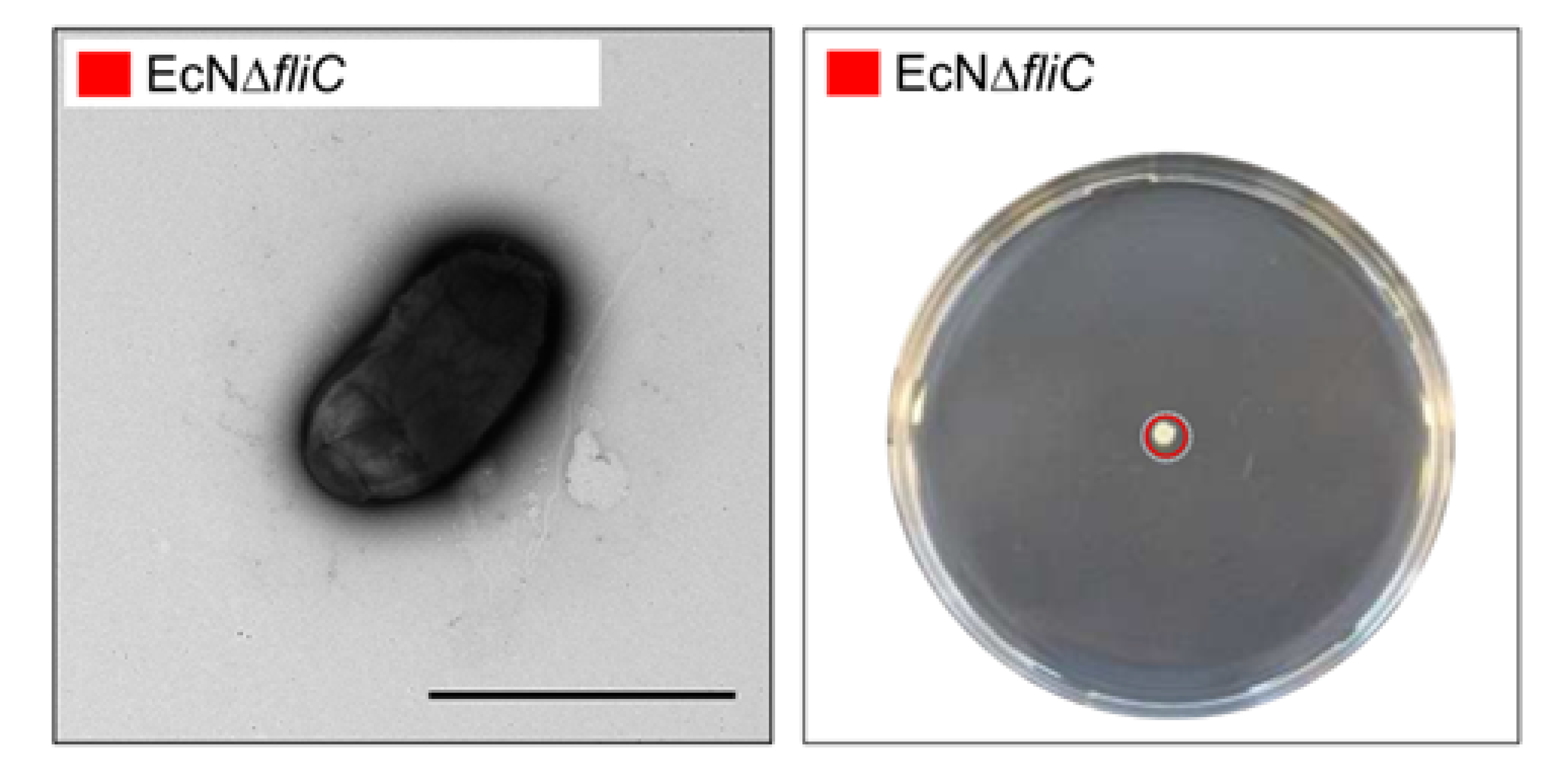

Supplement: S3 Fig — Right: overnight bacterial culture of EcNΔfliC was seeded in the middle of a swarming culture medium and incubated for 24 h. The inoculation spot is indicated by a red circle, and the borders of the swarming area are highlighted with a white scattered line. Left column: electron microscopy pictures (negative staining) of EcNΔfliC (highlighting the absence of flagella). EcN, E. coli Nissle 1917; fliC, flagellin. (PNG) [file pbio.3000334.s003.png]

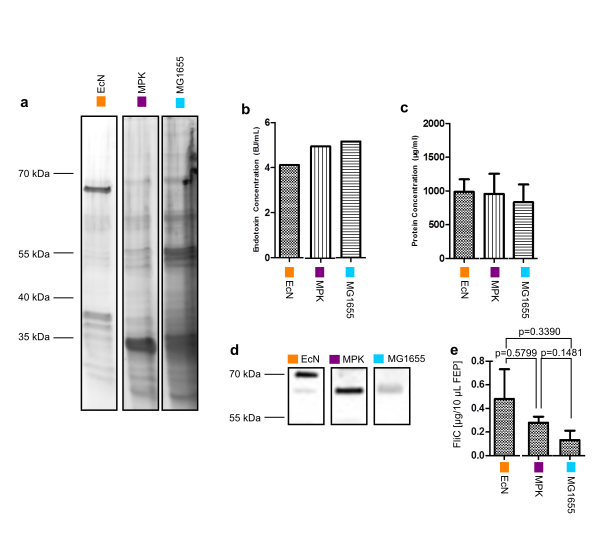

Supplement: S4 Fig — EcN, MPK, and MG1655 were grown to OD100, and FEPs were generated as described in the main manuscript. (a) Silver staining of 10 μL FEP on an 8%–15% gradient SDS gel. (b) LAL test to determine endotoxin levels in FEPs. (c) Determination of overall protein concentration in FEPs using a bicinchoninic acid assisted assay. (d) Western blot of FliC of 10 μL FEPs using anti-flagellin antibody (ab93713; Abcam, Cambridge, UK). (e) Quantification of FliC concentrations in FEPs. Band intensities of FliC bands in western blots depicted in (d) were quantified. A standard curve of recombinant EcN FliC was generated and visualized with the same antibodies on the same blots. FliC concentrations were computed using the determined FliC band intensities in relation to a linear regression of the band intensities of FliC standard curve. (b + c + e) The data underlying this figure can be found in S1 Data. EcN, E. coli Nissle 1917; FEP, flagella-enriched preparation; fliC, flagellin; LAL, limulus amebocyte lysate; MG1655, E. coli K12 MG1655; MPK, E. coli mpk; OD, optical density. (PNG) [file pbio.3000334.s004.png]

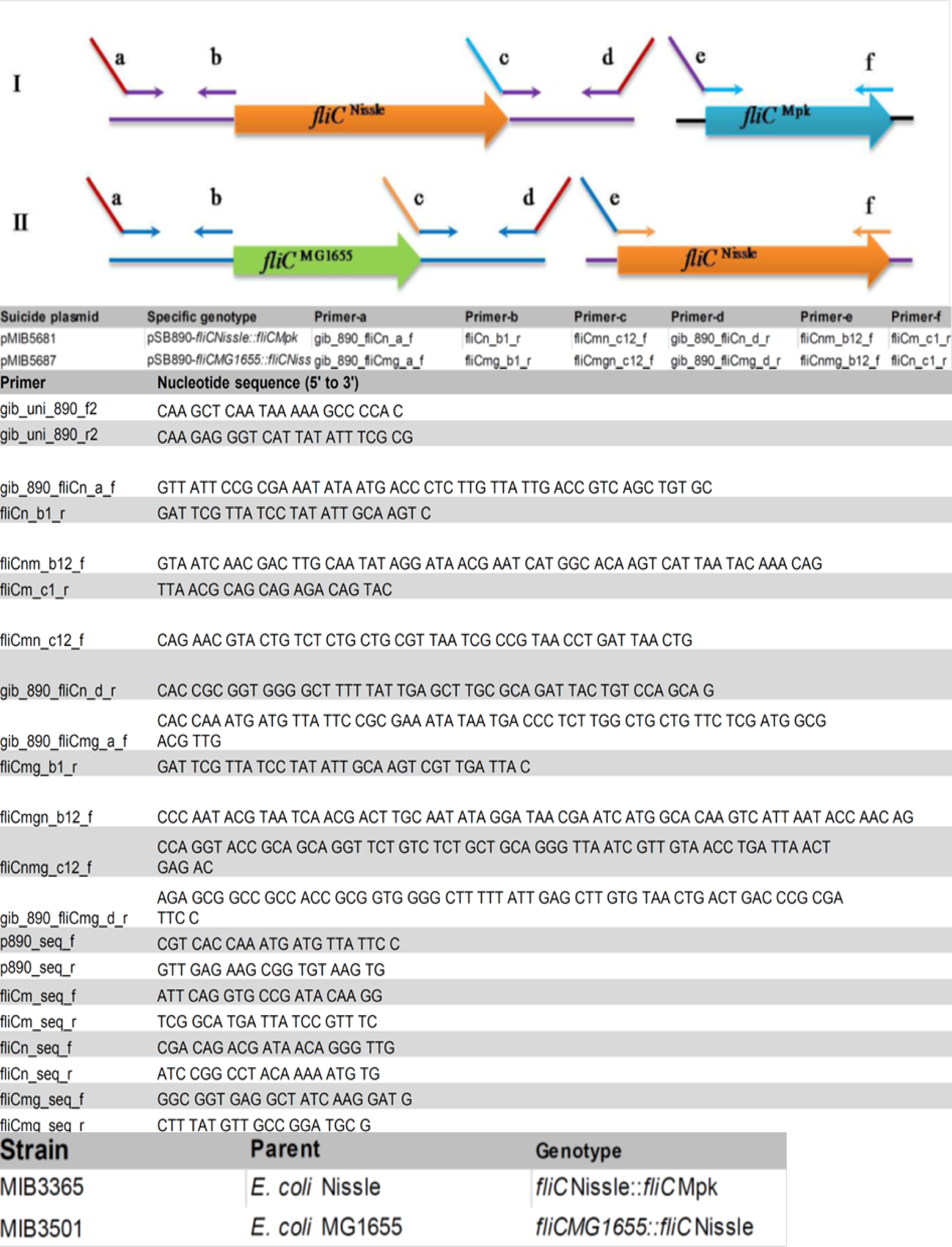

Supplement: S5 Fig — Chromosomal exchange of fliC alleles was done by allelic exchange as described previously [90]. Upper panel: suicide plasmids were constructed by Gibson assembly according to standard protocols [91]. Lower panel: primers and plasmids for allelic exchange as well as resulting strains. fliC, flagellin. (PNG) [file pbio.3000334.s005.png]

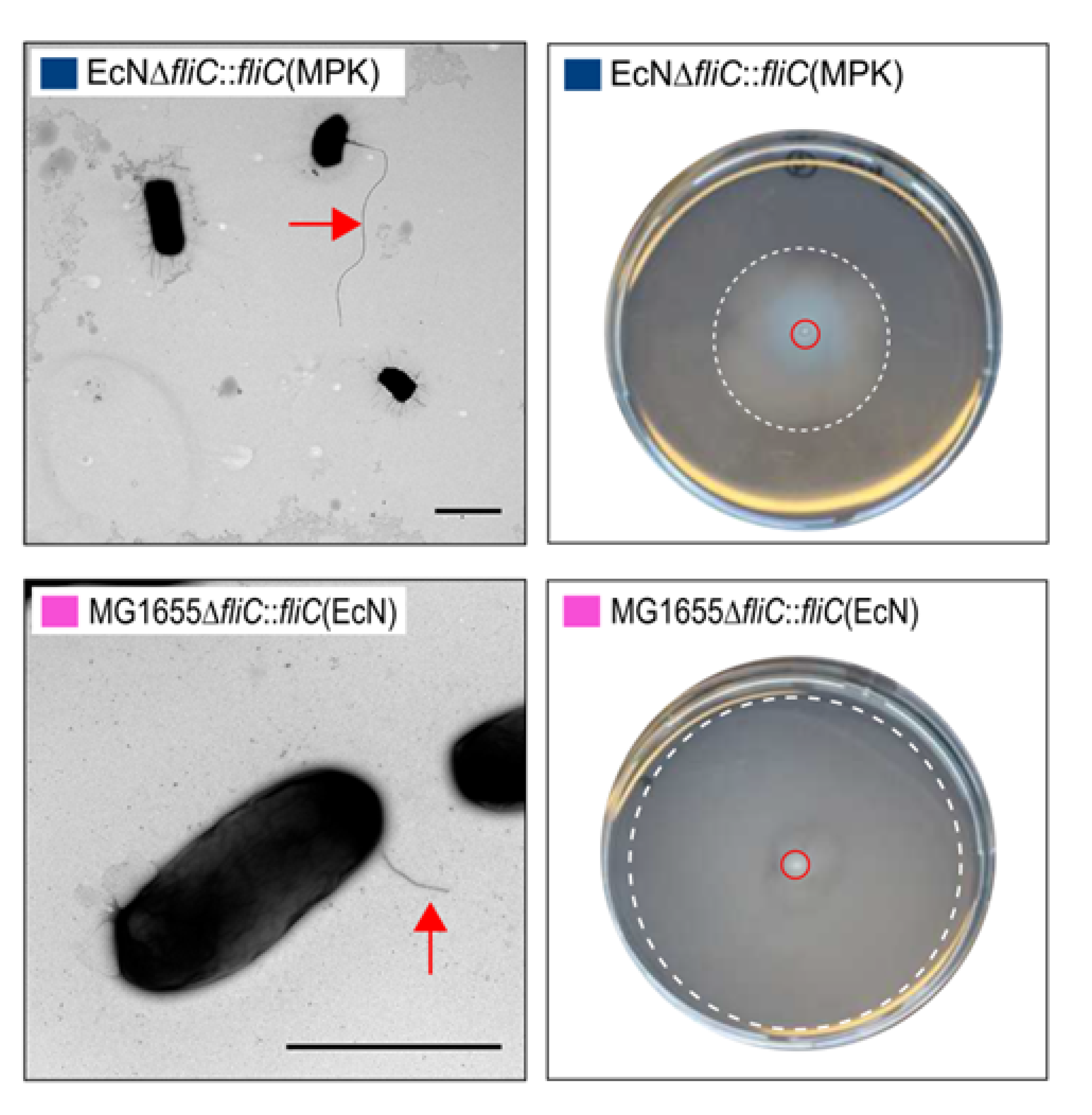

Supplement: S6 Fig — Right column: overnight bacterial culture of MG1655ΔfliC::fliC(EcN) and EcNΔfliC::fliC(MPK) exchange mutants were seeded in the middle of a swarming culture medium and incubated for 24 h. The inoculation spot is indicated by a red circle, and the borders of the swarming area are highlighted with a white scattered line. Left column: electron microscopy pictures of MG1655ΔfliC::fliC(EcN) and EcNΔfliC::fliC(MPK) highlighting the respective flagella (red arrow). EcN, E. coli Nissle 1917; fliC, flagellin; MG1655, E. coli K12 MG1655; MPK, E. coli mpk. (PNG) [file pbio.3000334.s006.png]

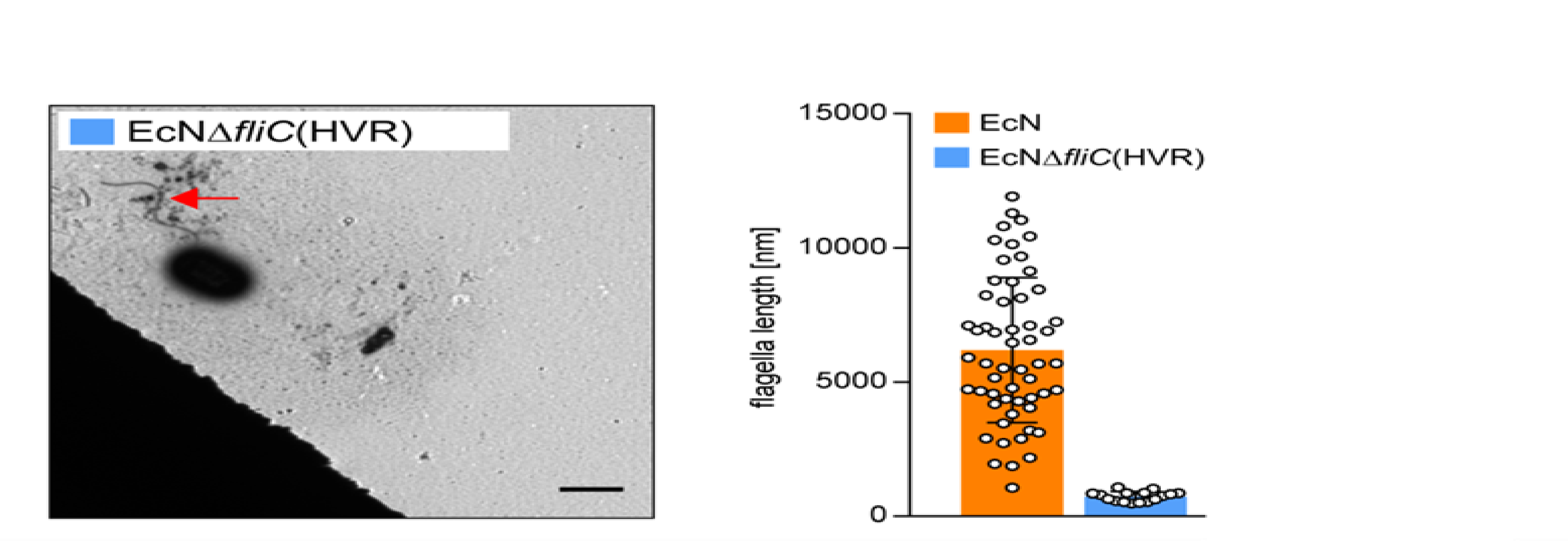

Supplement: S7 Fig — Left panel: EM pictures of EcNΔfliC(HVR) deletion mutants highlighting the flagella (red arrow). Right panel: EM-assisted determination of flagella lengths. Each white dot represents one detected flagellum in EM pictures. The data underlying this figure can be found in S1 Data. EcN, E. coli Nissle 1917; EM, electron microscopy; fliC, flagellin; HVR, hypervariable region; WT, wild type. (PNG) [file pbio.3000334.s007.png]

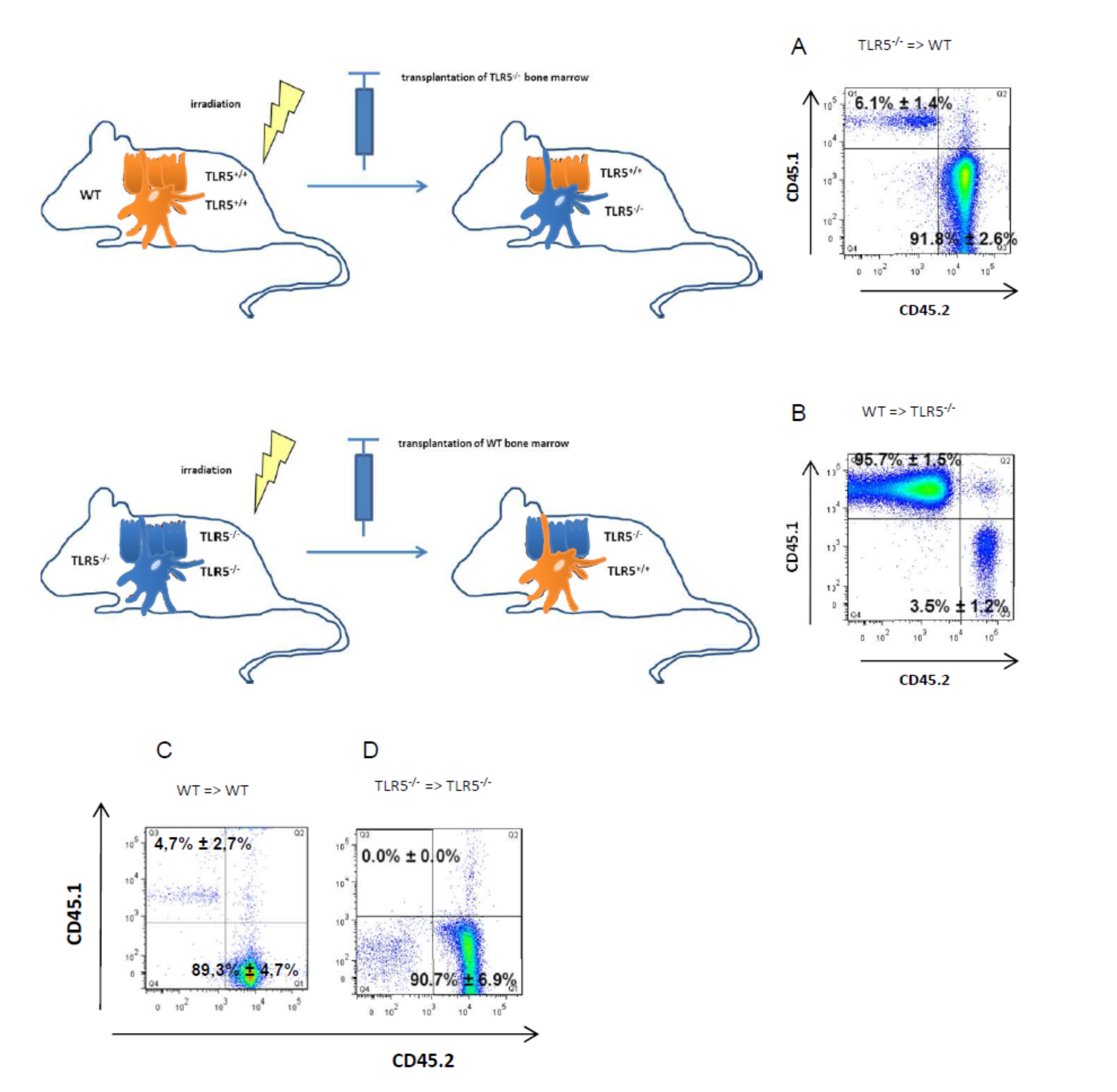

Supplement: S8 Fig — (A) Tlr5−/− → WT mice by irradiation of C57BL/6×WT-CD45.1–expressing mice transplanted with bone marrow of C57BL/6×Tlr5−/−-CD45.2–expressing mice and (B) WT → Tlr5−/− mice by irradiation of C57BL/6×Tlr5−/−-CD45.2–expressing mice transplanted with bone marrow of C57BL/6×WT-CD45.1–expressing mice. (C) Irradiated C57BL/6-CD45.2 mice transplanted with C57BL/6-CD45.1 bone marrow (WT → WT), (D) Tlr5−/−CD45.2 mice transplanted with Tlr5−/−-CD45.2 bone marrow (Tlr5−/− → Tlr5−/−). Successful transplantation was monitored by flow cytometry analysis of blood samples stained with antibodies against CD45.1 and CD45.2. Figures show means ± SD of 4 to 9 mice per experiment. BMCM, bone-marrow–chimeric mice; TLR, Toll-like receptor; WT, wild type. (PNG) [file pbio.3000334.s008.png]

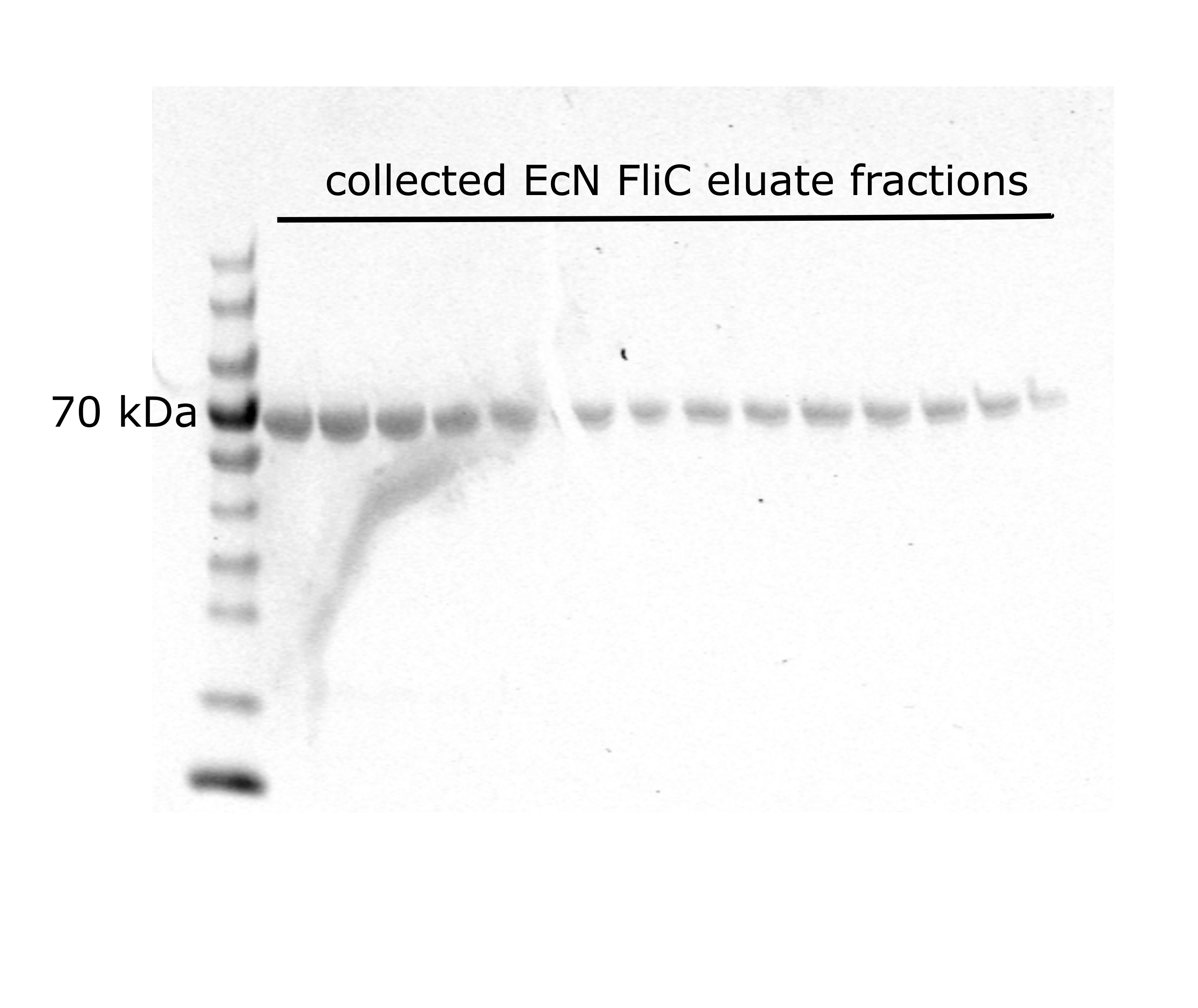

Supplement: S9 Fig — Coomassi-stained 4%–15% gradient gel of all collected eluted fractions after elution from HisTrap columns using elution buffer (300 mM NaCl, 50 mM Tris-HCl [pH 8.0], 500 mM imidazole) at concentrations from 20% to 100%. EcN, E. coli Nissle 1917; fliC, flagellin; rfliC(EcN), recombinant flagellin from EcN. (PNG) [file pbio.3000334.s009.png]

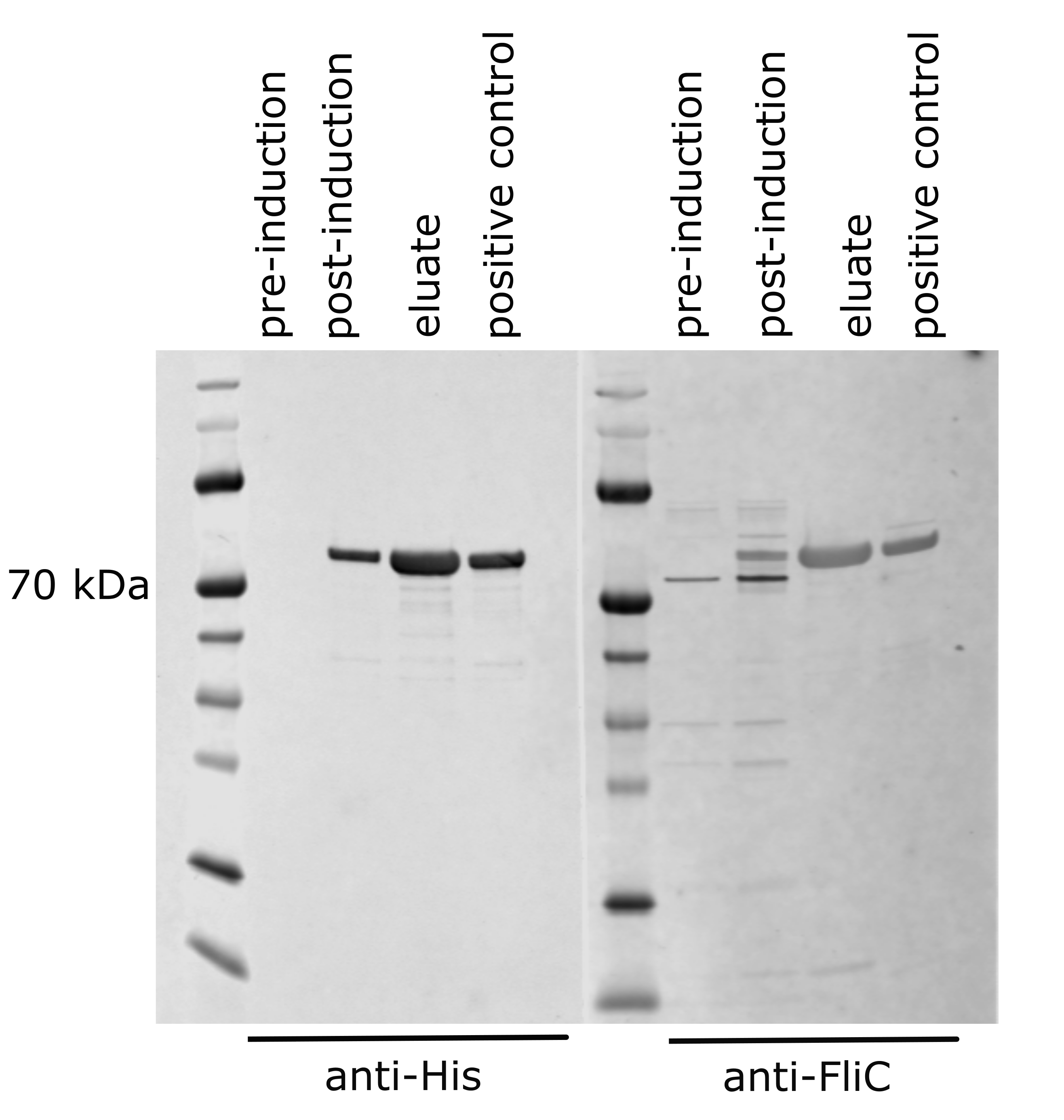

Supplement: S10 Fig — Western blots against the His-tags of rFliC(EcN) (left panel) and FliC (right panel) were performed to verify the proper expression of the recombinant protein. Cell lysates before IPTG-assisted induction of protein expression (preinduction), after IPTG-assisted induction (postinduction), the collected elutes from the HisTrap column (Elute) (see S9 Fig), and a previously purified MS-controlled rFliC(EcN) as positive control were loaded on a 4%–15% gradient gel, and western blots were performed as described. EcN, E. coli Nissle 1917; fliC, flagellin; MS, mass spectrometry; rfliC(EcN), recombinant flagellin from EcN. (PNG) [file pbio.3000334.s010.png]
