# Supplementary material for: Flagellin hypervariable region determines symbiotic properties of commensal Escherichia coli strains
Source: PLoS Biol. 2019 Jun 17;17(6):e3000334. doi: 10.1371/journal.pbio.3000334 (PMC6597123; doi:10.1371/journal.pbio.3000334)
Supplement: S1 Table — (DOCX) [file pbio.3000334.s011.docx]

**S1 Table**

Detailed statistical analysis of the values depicted in Fig 1e as determined by one-way ANOVA

| Comparison | Mean difference | Summary | p-value |
| --- | --- | --- | --- |
| WT DSS only vs. KO DSS only | 0,4485 | ns | 0,2148 |
| WT DSS only vs. WT DSS+EcN∆fliC | 1,636 | **** | <0,0001 |
| WT DSS only vs. KO DSS+EcN∆fliC | 0,8235 | * | 0,0103 |
| WT DSS only vs. WT DSS+EcN∆fliC | 0,3791 | ns | 0,4921 |
| WT DSS only vs. KO fliC | 0,7958 | ** | 0,0059 |
| KO DSS only vs. WT DSS+EcN∆fliC | 1,188 | **** | <0,0001 |
| KO DSS only vs. KO DSS+EcN∆fliC | 0,375 | ns | 0,6538 |
| KO DSS only S vs. WT fliC | -0,06944 | ns | 0,9996 |
| KO DSS only vs. KO fliC | 0,3472 | ns | 0,6551 |
| WT DSS+EcN vs. KO DSS+EcN | -0,8125 | * | 0,0415 |
| WT DSS+EcN vs. WT DSS+EcN∆fliC | -1,257 | **** | <0,0001 |
| WT DSS+EcN vs. KO DSS+EcN∆fliC | -0,8403 | * | 0,0182 |
| KO DSS+EcN vs. WT DSS+EcN∆fliC | -0,4444 | ns | 0,5383 |
| KO DSS+EcN vs. KO DSS+EcN∆fliC | -0,02778 | ns | >0,9999 |
| WT DSS+EcN∆fliC vs. KO DSS+EcN∆fliC | 0,4167 | ns | 0,5359 |
