# Supplementary material for: Flagellin hypervariable region determines symbiotic properties of commensal Escherichia coli strains
Source: PLoS Biol. 2019 Jun 17;17(6):e3000334. doi: 10.1371/journal.pbio.3000334 (PMC6597123; doi:10.1371/journal.pbio.3000334)
Supplement: S2 Table — p-values were computed using nonparametric Kruskal–Wallis test. DSS, dextran sodium sulphate. (DOCX) [file pbio.3000334.s012.docx]

**S2 Table**:

Statistical analysis of cytokine serum levels in DSS-treated mice

|  | Group 1:  DSS (n = 4) | | Group 2:  DSS + EcN (n = 9) | | Group 3:  DSS + EcN∆flic(HVR)  (n = 5) | | Group 1 vs Group 2 | Group 1 vs Group 3 | Group 2 vs Group 3 |
| --- | --- | --- | --- | --- | --- | --- | --- | --- | --- |
| Cytokine | Mean | SD | Mean | SD | Mean | SD | p | p | p |
| **IL-10** | 91.0 | 23.25 | 55.2 | 9.2 | 105.2 | 49.5 | 0.0017 * | 0.6162 | 0.0104 * |
| **IL-1β** | 38.75 | 2.2 | 35.3 | 0.8 | 45.2 | 15.3 | 0.0016 * | 0.4372 | 0.0694 |
| IFNγ | 28.0 | 7.0 | 21.6 | 2.3 | 54.3 | 66.6 | 0.0321 * | 0.4626 | 0.1467 |
| IL-12p70 | 173.1 | 10.8 | 166.9 | 14.6 | 180.0 | 21.1 | 0.4200 | 0.6092 | 0.1920 |
| **IL-23** | 335.0 | 315.8 | 117.7 | 20.9 | 172.6 | 58.9 | 0.0519 | 0.2900 | 0.0239 * |
| IL-1α | 30.0 | 14.5 | 26.3 | 13.2 | 29.4 | 13.7 | 0.6580 | 0.9510 | 0.6840 |
| TNFα | 37.0 | 7.8 | 30.1 | 2.3 | 29.8 | 2.6 | 0.0328 * | 0.0901 | 0.8400 |
| **IL-6** | 69.3 | 30.6 | 30.6 | 8.6 | 45.0 | 15.4 | 0.0062 * | 0.1628 | 0.0513 |
| **IL-27** | 352.5 | 232.4 | 166.0 | 11.1 | 216.8 | 50.9 | 0.0270 * | 0.2383 | 0.0119 * |
| **IL-17A** | 12.7 | 2.7 | 6.3 | 2.0 | 12.4 | 5.4 | 0.0009 * | 0.9110 | 0.0136 * |
| **IFNβ** | 92.2 | 48.1 | 51.2 | 11.4 | 163.8 | 111.4 | 0.0278 * | 0.2744 | 0.0091 * |
| GM-CSF | 356.3 | 19.5 | 361.2 | 93.4 | 374.4 | 21.8 | 0.9198 | 0.2357 | 0.7650 |
| MCP-1 | 431.0 | 86.2 | 369.6 | 93.2 | 383.2 | 24.0 | 0.2870 | 0.2688 | 0.7573 |
