# Supplementary material for: Flagellin hypervariable region determines symbiotic properties of commensal Escherichia coli strains
Source: PLoS Biol. 2019 Jun 17;17(6):e3000334. doi: 10.1371/journal.pbio.3000334 (PMC6597123; doi:10.1371/journal.pbio.3000334)
Supplement: S1 Text — (DOCX) [file pbio.3000334.s014.docx]

**Title**

**Flagellin hypervariable region determines symbiotic properties of commensal *Escherichia coli* strains**

**Authors**

Alex Steimle^1,5^*, Sarah Menz^1,5^*, Annika Bender^1,5^, Brianna Ball^1,5^, Alexander N. R. Weber^2^, Sina Beier^3^, Mehari Tesfazgi Mebrhatu^1,5^, Kerstin Gronbach^1,5^, Samuel Wagner^1,5^, David Voehringer^4^, Anna Lange^1,5^, Martin Schaller^6^, Birgit Fehrenbacher^6^, Ingo B. Autenrieth^1,5^, Tobias A. Oelschlaeger^4^ and Julia-Stefanie Frick^1,4#^

^1^Institute of Medical Microbiology and Hygiene, University of Tübingen, Tübingen, Germany

^2^Department of Immunology, University of Tübingen, Tübingen, Germany

^3^Chair of Algorithms in Bioinformatics, Faculty of Computer Science, University of Tübingen, Tübingen, Germany

^4^Department of Infection Biology, University Hospital Erlangen, Erlangen, Germany

^5^German Center for Infection Research (DZIF), Partner Site Tübingen, Tübingen, Germany

^6^Department of Dermatology, University Hospital Tübingen, Tübingen, Germany

* These authors contributed equally

^#^ corresponding author

**Short title**

Flagellin structure determines immunogenicity of *E. coli* strains

**Supporting information**

**Supplementary methods**

**Isolation of lamina propria (LP) cells**

Isolation of lp cells was performed as published previously [1].

**Flow cytometry**

Flow cytometry was used to assess reconstitution of transplanted bone marrow cells and to characterize lamina propria (lp) CD11c^+^ cells. Cells were stained in PBS + 1% FCS with the following antibodies: anti-CD3 (clone 145-2C11), anti-CD4 (RM4-5), anti-CD11c (HL3), anti-TGFβ (TW7-16B4), anti-CD62L (MEL-14), anti-CD45.1 (A20) and anti-CD45.2 (104). All antibodies were purchased from BD Biosciences, Germany. Cells were washed and fixed in 100 µL PBS +1% FCS and 50 µL 4% formalin. Analyses were performed on a FACS LSRII or FACS Calibur (BD Biosciences). Data were analyzed using the FlowJo software 7.6.1 (Tree Star Inc., USA). Cell viability was assessed by using BD VS450 Fixable Viability stain according to the manufacturer’s instruction.

**Intracellular cell staining**

2 x 10^6^ cells were incubated in DMEM (Gibco) supplemented with 10% FCS, 1% Hepes, 1% non-essential amino acids, 1% sodium pyruvat, 0.5% penicillin/streptomycin, 0.5% ß-mercaptoethanol and 2 µL leukocyte activation cocktail (BD Biosciences) for 4h at 37°C. Cells were washed and fixed with Cytofix / Cytoperm (BD Biosciences). After washing with PBS + 10 % Saponin + 3% FCS cells were stained.

**Culture of mouse TLR5 expressing human embryonic kidney cells and detection of IL-8 secretion in response to TLR5 receptor activation**

Mouse TLR5 expressing human embryonic kidney 293 cells (mTLR5-HEK293 cells) were cultured in DMEM supplemented with 10% FCS and 1% penicillin/streptomycin. 2 x 10^5^ cells were stimulated with bacteria or bacterial FEP for 24 h as indicated in the results section. PBS was used as a negative control. IL-8 concentration in supernatants were determined by ELISA (BD Biosciences) according to the manufacturer’s instructions.

**Amino acid sequence alignment**

By using MAFFT an amino acid sequence alignment was performed of the following flagellins: *E. coli* Nissle, *E. coli* MG1655 and *E. coli* mpk.

**Negative Staining Transmission Electron Microscopy**

Suspension (Bacteria) were placed directly onto a glow-discharged EM grid. After adsorption, the grids were washed in double distilled water and negatively stained with 1% uranyl acetate. The grids were examined using a Zeiss LIBRA 120 transmission electron microscope (Carl Zeiss Oberkochen, Germany) operating at 120 kV.

**Transmission Electron Microscopy**

Bacteria were fixed with Karnovsky’s fixative for 24h at 4°C. Post-fixation was based on 1.0% osmium tetroxide containing 1.5% K-ferrocyanide in 0,1M cacodylate buffer for 2 h.

After following standard methods, blocks were embedded in glycide ether and cut using an ultra microtome (Ultracut, Reichert, Vienna, Austria). Ultra-thin sections (30 nm) were mounted on copper grids and analyzed using a Zeiss LIBRA 120 transmission electron microscope (Carl Zeiss, Oberkochen, Germany) operating at 120 kV

**Western Blotting**

For western blot analysis of fliC, antibody Ab93713 (abcam) was used. FEP were generated as described and 10 µL FEPs were transferred on 8%-15% gradient polyacrylamide gels. Proteins were separated via SDS-PAGE gels and transferred to nitrocellulose membranes. Membranes were blocked subsequently for 1h at room temperature in a BSA containing blocking buffer (LiCor) followed by incubation with primary Abs diluted 1:1000 in blocking buffer over night at 4°C. After incubation the membranes were washed three times in PBS/T (PBS pH 7.4 + 0.1% Tween-20) and were subsequently incubated with fluorochrome coupled secondary antibodies (LiCor) according to the manufacturer’s instruction for 2h at room temperature. After repeating the washing step with PBS/T the membranes were washed twice with PBS to remove detergent from the membranes. Proteins were detected using the LiCor visualization system.

**Histology**

Colon tissues were fixed in neutral buffered 4% formalin. Formalin-fixed tissues were embedded in paraffin and cut in 2 µm sections. Samples were stained with hematoxylin and eosin (Merck). Sections were analyzed in a blinded fashion. Scores (0-3) were given for two different parameters: 1) Severity of inflammation: rare inflammatory cells in the lamina propria (0); increased numbers of inflammatory cells (1); confluence of inflammatory cells extending into the submucosa (2); and transmural extension of the inflammatory cell infiltrate (3). 2) Extent of injury: no mucosal damage (0); discrete lymphoepithelial lesions (1); surface mucosal erosion (2); and widespread mucosal ulceration and extension through deeper bowel wall structures (3). The scores of the two parameter were added, and the mean was calculated.

**Cytokine detection in mouse blood serum**

Blood was collected from the heart of sacrificed mice. Blood samples were stored on ice for 15 min and subsequently centrifuged for 10 min at 1’000 x g. Serum was collected and stored at -80 °C until analysis. Serum concentrations of 13 different cytokines were detected using the Biolegend Legendplex Immunoassay according to the manufacturer’s instructions. Serum samples were used undiluted for this approach. Cytokine concentrations were computed using internal standards and with the help of a 4-parameter logarithmic standard curve provided by elisaanalysis.com.

References

1. Jeon SG, Kayama H, Ueda Y, et al. Probiotic Bifidobacterium breve induces IL-10-producing Tr1 cells in the colon. PLoS Pathog 2012;8:e1002714.
